# Genetic Parameters and Genome-Wide Association Studies of Eight Longevity Traits Representing Either Full or Partial Lifespan in Chinese Holsteins

**DOI:** 10.3389/fgene.2021.634986

**Published:** 2021-02-25

**Authors:** Hailiang Zhang, Aoxing Liu, Yachun Wang, Hanpeng Luo, Xinyi Yan, Xiangyu Guo, Xiang Li, Lin Liu, Guosheng Su

**Affiliations:** ^1^Key Laboratory of Animal Genetics, Breeding and Reproduction, MARA, National Engineering Laboratory of Animal Breeding, College of Animal Science and Technology, China Agricultural University, Beijing, China; ^2^Center for Quantitative Genetics and Genomics, Aarhus University, Tjele, Denmark; ^3^Beijing Dairy Cattle Center, Beijing, China

**Keywords:** lifespan, heritability, genetic correlation, candidate gene, dairy cattle

## Abstract

Due to the complexity of longevity trait in dairy cattle, two groups of trait definitions are widely used to measure longevity, either covering the full lifespan or representing only a part of it to achieve an early selection. Usually, only one group of longevity definition is used in breeding program for one population, and genetic studies on the comparisons of two groups of trait definitions are scarce. Based on the data of eight traits well representing the both groups of trait definitions, the current study investigated genetic parameters and genetic architectures of longevity in Holsteins. Heritabilities and correlations of eight longevity traits were estimated using single-trait and multi-trait animal models, with the data from 103,479 cows. Among the cows with phenotypes, 2,630 cows were genotyped with the 150K-SNP panel. A single-trait fixed and random Circuitous Probability Unification model was performed to detect candidate genes for eight longevity traits. Generally, all eight longevity traits had low heritabilities, ranging from 0.038 for total productive life and herd life to 0.090 for days from the first calving to the end of first lactation or culling. High genetic correlations were observed among the traits within the same definition group: from 0.946 to 0.997 for three traits reflecting full lifespan and from 0.666 to 0.997 for five traits reflecting partial productive life. Genetic correlations between two groups of traits ranged from 0.648 to 0.963, and increased gradually with the extension of lactations number regarding the partial productive life traits. A total of 55 SNPs located on 25 chromosomes were found genome-wide significantly associated with longevity, in which 12 SNPs were associated with more than one trait, even across traits of different definition groups. This is the first study to investigate the genetic architecture of longevity representing both full and the partial lifespan simultaneously, which will assist the selection of an appropriate trait definition for genetic improvement of longevity. Because of high genetic correlations with the full lifespan traits and higher heritability, the partial productive life trait measured as the days from the first calving to the end of the third lactation or culling could be a good alternative for early selection on longevity. The candidate genes identified by this study, such as RPRM, GRIA3, GTF2H5, CA5A, CACNA2D1, FGF10, and DNAJA3, could be used to pinpoint causative mutations for longevity and further benefit the genomic improvement of longevity in dairy cattle.

## Introduction

Longevity is an economically important trait in dairy cattle, due to its large impact on the efficiency of dairy farming (Weigel et al., 1995; Essl, 1998). The improvement of longevity allows a farm to have not only a higher voluntary culling rate but also a lower involuntary culling rate. On one hand, by reducing involuntary culling rate, the extra costs used for the replacement heifers can be reduced (Jairath et al., 1994; Veerkamp et al., 1995; Brotherstone et al., 1997; Essl, 1998). On the other hand, genetic improvements of a population can be speeded up due to the possibility to increase voluntary culling rate while keep a relatively constant population size (Essl, 1998).

Since 1990s, longevity has been included in the total selection index in many countries (Interbull^1^) (Miglior et al., 2017). For example, by giving 5 ∼ 14% weights to productive life (covering the full lifespan trait) in the selection index, the longevity in United States Holsteins has started to improve. However, the longevity traits representing the full lifespan is only available after the individual being culled or dead, and thus, the selection response of longevity trait was slow down by the balance between a long generation interval (collecting large scale phenotype) and low selection accuracy (data available). An early selection of longevity could be achieved by using traits which measure a partial lifespan, such as the days from the first calving to a certain lactation. For example, a total of 5 traits including productive days during period from first calving to the end of the first (Lon11), second (Lon12), third (Lon13), fourth (Lon14), or fifth lactation (Lon15) were used to evaluate longevity in the Nordic Cattle Genetic Evaluation^2^. Despite the potential to improve longevity genetically, in Chinese Holsteins, the selection for longevity traits has not yet been implemented. The investigation of genetic parameters for longevity traits with different definitions is critical for the selection of proper longevity traits to be added to the selection index [i.e., China Dairy Performance Index (CPI)] (Dairy Association of China, Beijing^3^) for the genetic improvement of longevity in Chinese Holsteins. A large number of studies on genetic analysis of longevity were performed in various dairy cattle populations, but all studies only investigated one of the two groups of trait definition, either full lifespan or partial lifespan (Tsuruta et al., 2005; Sewalem et al., 2007; van Pelt et al., 2015; Clasen et al., 2017; Imbayarwo-Chikosi et al., 2017) and none of them ever explored the genetic relationships among longevity traits with different definitions.

By incorporating the information of genetic makers associated with the target trait into selection decisions, faster genetic progress could be achieved (Liu et al., 2017, 2020). With the help of single nucleotide polymorphism (SNP) panel, genome-wide association studies (GWAS) have been used as primary strategies to identify genetic makers for complex traits since Klein (2005). For longevity traits, GWAS have been performed for productive life (Cole et al., 2011; Nayeri et al., 2017) and herd life in United States Holsteins (Nayeri et al., 2017), for productive life and herd life in Italy Holsteins (Steri et al., 2019), for productive life in Thai crossbred Holsteins (Saowaphak et al., 2017), German-Austrian Fleckvieh (Mészáros et al., 2014), and United States composite beef breeds (1/2 Red Angus, 1/4 Charolais, 1/4 Tarentaise) (Hay and Roberts, 2017), and for partial productive life in Nordic Holsteins, Red cattle and Jersey (Zhang et al., 2016). Nevertheless, the genetic markers of longevity obtained from various GWAS did not overlap well across populations. In addition to the possible differences in linkage disequilibrium (LD) structures across populations and the insufficient detection power due to low heritabilities, the different trait definitions being used in different studies could also be a reason.

The objectives of this study were (1) to estimate heritabilities and genetic correlations for longevity traits with different definitions, including both traits representing full lifespan and traits representing partial lifespan; and (2) to identify genetic variants associated with longevity. Results of this study will assist the selection of appropriate trait definitions to be used for genetic improvement of longevity in dairy cattle.

## Results

### Longevity in Chinese Holsteins

The descriptive statistics of longevity traits in the Chinese Holstein population are presented in Table 1, and the distributions of each longevity trait is presented in Supplementary Figure 1. The Chinese Holstein cows had 2.7 ± 1.6 lactations in average. In line with the definitions, the length in days of full lifespan traits (from 822 to 1, 618 days) were larger than the partial productive life, except for the milking life (738 days). The coefficient of variation for partial lifespan traits increased gradually with the extension of number of lactations, and the coefficients of variation for full lifespan traits were larger than that for partial lifespan traits, except for herd life. With the extension of the number of lactations, the average partial productive life change from 331 (Lon11) to 749 days (Lon15). However, the magnitude of increase in partial productive life with increasing number of lactations decreased gradually. For example, the increase of productive life was 231 day when increasing from Lon11 to Lon12, but only 18 days when increasing from Lon 14 to Lon15. The change of phenotype value from Lon 11 to Lon15 reflects both individual variation for real longevity and impacts from censored records.

**TABLE 1:** Descriptive statistics of cow longevity traits in Chinese Holsteins.

Traits^1^	N	Min	Max	Mean	SD	Coefficient of variation
Lon11	103,479	1	365	330.6	93.2	28.2
Lon12	90,279	1	730	560.9	235.3	42.0
Lon13	82,826	1	1,095	680.9	368.2	54.1
Lon14	79,571	1	1,460	731.4	458.7	62.7
Lon15	78,144	1	1,825	749.2	510.0	68.1
ML	78,227	1	3,836	738.0	506.8	68.7
PL	78,227	1	4,007	822.3	575.9	70.0
HL	78,227	1	4,825	1,617.7	588.7	36.4
Lactation	117,491	1	13	2.7	1.6	60.2

*^1^Lon11, the days from the first calving to the end of the first lactation or culling; Lon12, the days from the first calving to the end of the second lactation or culling; Lon13, the days from the first calving to the end of the third lactation or culling; Lon14, the days from the first calving to the end of the fourth lactation or culling; Lon15, the days from the first calving to the end of the fifth lactation or culling; PL, productive life referring the days from the first calving to culling or death; ML, milking life referring the days from the first calving to culling or death but excludes all dry periods; HL, herd life referring the days from birth to culling or death; Lactation, number of lactations at culling or death.*

### Heritabilities and Genetic Correlations

The estimates of variance component and heritability for eight longevity traits from single-trait animal models are shown in Table 2. Generally, the partial productive life traits had higher heritabilities (ranged from 0.051 for Lon14 to 0.090 for Lon11) than those for the full lifespan traits (ranged from 0.038 for PL and HL to 0.040 for ML). Among the five partial productive life traits, heritabilities decreased with the increase of number of lactations. Among the three full lifespan traits, ML had the highest heritability. Standard errors of heritabilities were low (lower than 0.010), suggesting that all the heritabilities were accurately estimated.

**TABLE 2:** Estimates of additive genetic variance (${\hat{\sigma }}_{\text{a}}^{2}$), residual variance (${\hat{\sigma }}_{\text{e}}^{2})$, and heritability (${\hat{\text{h}}}^{2})$ for eight longevity traits in Chinese Holsteins.

Trait^1^	N	${\hat{\sigma }}_{a}^{2}$	${\hat{\sigma }}_{e}^{2}$	${\hat{\text{h}}}^{2}$
Lon11	103,479	725.13 ± 64.05	7,333.92 ± 58.52	0.090 ± 0.008
Lon12	90,279	2,812.37 ± 292.38	42,117.79 ± 301.72	0.063 ± 0.006
Lon13	82,826	5,557.25 ± 610.59	98,433.22 ± 680.48	0.053 ± 0.006
Lon14	79,571	8,111.88 ± 903.21	151,005.01 ± 1,038.21	0.051 ± 0.006
Lon15	78,144	10,476.65 ± 1157.65	186,076.45 ± 1,309.04	0.053 ± 0.006
ML	78,227	7,811.06 ± 995.05	187,066.34 ± 1,237.10	0.040 ± 0.005
PL	78,227	9,627.98 ± 1,236.02	241,743.18 ± 1,574.84	0.038 ± 0.005
HL	78,227	9,602.13 ± 1,232.00	241,404.44 ± 1,571.52	0.038 ± 0.005

*^1^Lon11, the days from the first calving to the end of the first lactation or culling; Lon12, the days from the first calving to the end of the second lactation or culling; Lon13, the days from the first calving to the end of the third lactation or culling; Lon14, the days from the first calving to the end of the fourth lactation or culling; Lon15, the days from the first calving to the end of the fifth lactation or culling; PL, productive life referring the days from the first calving to culling or death; ML, milking life referring the days from the first calving to culling or death but excluding all dry periods; HL, herd life referring the days from birth to culling or death.*

Genetic and phenotypic correlations among HL, PL, and ML and among Lon11, Lon12, Lon13, Lon14, and Lon15 estimated using multi-trait animal models are presented in Table 3. Genetic correlations among three traits representing the full lifespan were close to the unity and with standard errors lower than 0.01. For any of the two partial productive life traits, the traits with the closest lactation number usually had higher genetic correlations. For example, the genetic correlation between Lon11 and Lon12 was 0.912, while genetic correlations between Lon11 and Lon13, Lon14, and Lon15 were 0.774, 0.698, and 0.666, respectively. For the partial productive life traits with a period at least 2 lactations (including Lon12, Lon13, Lon14, and Lon15), genetic correlations among these traits were higher than 0.9, ranging from 0.922 between Lon12 and Lon15 to 0.997 between Lon14 and Lon15. The genetic correlations between PL and various partial productive life traits increased gradually with increasing number of lactations of the partial productive life traits. For example, a very high genetic correlation was observed between PL and Lon15 (0.963 ± 0.007), whereas a moderate genetic correlation was observed between PL and Lon11 (0.648 ± 0.044).

**TABLE 3:** Genetic (r_g_) and phenotypic (r_p_) correlations between longevity traits in Chinese Holsteins.

Trait definition group	Trait1^1^	Trait2	rg ± SE	rp ± SE
Partial lifespan traits	Lon11	Lon12	0.912 ± 0.014	0.789 ± 0.072
	Lon11	Lon13	0.774 ± 0.031	0.622 ± 0.135
	Lon11	Lon14	0.698 ± 0.039	0.533 ± 0.167
	Lon11	Lon15	0.666 ± 0.042	0.487 ± 0.180
	Lon12	Lon13	0.981 ± 0.004	0.919 ± 0.033
	Lon12	Lon14	0.943 ± 0.011	0.831 ± 0.069
	Lon12	Lon15	0.922 ± 0.015	0.775 ± 0.089
	Lon13	Lon14	0.994 ± 0.002	0.964 ± 0.015
	Lon13	Lon15	0.984 ± 0.004	0.922 ± 0.033
	Lon14	Lon15	0.997 ± 0.001	0.984 ± 0.007
Full lifespan traits	HL	PL	0.951 ± 0.007	0.998 ± 0.001
	HL	ML	0.946 ± 0.008	0.994 ± 0.003
	PL	ML	0.997 ± 0.001	0.996 ± 0.002
Across trait groups	PL	Lon11	0.648 ± 0.044	0.462 ± 0.188
	PL	Lon12	0.865 ± 0.022	0.707 ± 0.114
	PL	Lon13	0.938 ± 0.011	0.842 ± 0.065
	PL	Lon14	0.958 ± 0.008	0.907 ± 0.040
	PL	Lon15	0.963 ± 0.007	0.937 ± 0.028

*^1^Lon11, the days from the first calving to the end of the first lactation or culling; Lon12, the days from the first calving to the end of the second lactation or culling; Lon13, the days from the first calving to the end of the third lactation or culling; Lon14, the days from the first calving to the end of the fourth lactation or culling; Lon15, the days from the first calving to the end of the fifth lactation or culling; PL, productive life referring the days from the first calving to culling or death; ML, milking life referring the days from the first calving to culling or death but excluding all dry periods; HL, herd life referring the days from birth to culling or death.*

### GWAS

The SNPs reached the genome-wide significant level in association with longevity traits in Chinese Holsteins are presented in Table 4. In total, 55 SNPs located on 25 chromosomes were genome-wide significantly associated with longevity traits. There were 12 SNPs significantly associated with more than one trait, in which 8 SNPs were shared among the traits within the same group of partial productive life and 4 SNPs were across two groups. For example, the SNP rs135565406 was significantly associated with HL, ML, PL, Lon12, and Lon15. In terms of chromosomes, the chromosome with most SNPs (9 SNPs) significantly associated with longevity traits was chromosome X. For each trait, the number of significantly associated SNPs range from 6 (ML) to 11 (HL, PL, or Lon12) with an average of 9.

**TABLE 4:** The genome-wide significant SNPs and candidate genes associated with longevity traits in Chinese Holsteins.

Chromosome	Position^1^(bp)	SNP	Trait^2^	MAF^3^	P-value	Effect size^4^ (%)	Candidate gene^5^
2	91,629,36	rs135750821	Lon15	0.43	1.71 E-07	0.49	CALCRL
2	42,630,124	rs137712544	Lon11	0.39	1.82 E-08	1.00	RPRM
2	46,201,858	rs110628337	Lon15	0.35	3.48 E-07	0.67	–
3	64,114,128	rs42867525	ML	0.33	2.96 E-09	0.73	–
3	118,421,054	rs137402734	Lon14	0.39	1.72 E-08	0.65	–
4	11,190,947	rs41613051	Lon11	0.36	5.72 E-08	0.58	TFPI2, GNGT1, GNG11, BET1
4	38,428,113	rs136492033	PL	0.36	1.28 E-07	0.58	CACNA2D1
4	85,565,338	rs109342356	ML	0.18	1.10 E-07	0.50	ING3
5	115,983,568	rs135565406	HL, ML, PL, Lon11, Lon15,	0.42	9.96 E-10, 3.20 E-09, 7.95 E-08, 4.34 E-08, 7.01 E-08	0.73, 0.74, 0.55, 0.66, 0.63	RIBC2, FBLN1, ATXN10
6	2,639,865	rs137700260	HL, Lon13, Lon14	0.07	3.42 E-07, 2.87 E-08, 1.35 E-07	0.47, 0.60, 0.54	NPY1R, NAF1
6	6,916,170	rs43451042	Lon15	0.43	2.69 E-08	0.46	PRSS12, NDST3
7	43,904,171	rs29012367	HL	0.36	1.27 E-08	0.53	CIRBP, FAM174C, PWWP3A, NDUFS7, GAMT, DAZAP1, RPS15, C7H19orf25, REEP6, MBD3, UQCR11, TCF3
7	54,635,988	rs132789189	HL	0.45	5.21 E-09	0.65	NR3C1
7	95,581,783	rs109221022	Lon15	0.39	4.11 E-07	0.68	PCSK1
8	72,165,064	rs43559099	Lon12	0.26	6.83 E-08	0.49	NEFM, NEFL
8	72,275,281	rs134248248	Lon13	0.43	3.68 E-07	0.70	NEFM, NEFL
9	15,157,597	rs42693004	Lon12	0.15	3.46 E-07	0.55	FILIP1, SENP6
9	94,882,463	rs134672623	Lon12, Lon15	0.45	2.59 E-07, 2.35 E-08	0.55, 0.95	SERAC1, GTF2H5, DYNLT1
9	99,144,354	rs109415769	Lon14	0.24	9.83 E-08	0.44	QKI
11	14,367,967	rs133167045	ML	0.17	8.37 E-09	0.53	XDH, SRD5A2, MEMO1
11	24,568,406	rs41592158	PL	0.38	3.61 E-07	0.42	PKDCC, EML4, COX7A2L
12	12,473,832	rs43691605	Lon13, Lon14	0.41	3.73 E-08, 2.14 E-08	0.48, 0.53	–
12	18,879,230	rs109505275	Lon11	0.30	9.14 E-09	0.80	FNDC3A, CDADC1, SETDB2, PHF11
13	71,211,797	rs109520811	PL	0.19	2.99 E-10	0.94	–
14	9,447,596	rs110760208	PL	0.15	3.16 E-08	0.61	–
14	11,909,376	rs110417753	HL	0.29	3.94 E-08	0.65	–
14	17,597,579	rs110392124	HL	0.35	8.15 E-08	0.53	–
14	52,854,862	rs133758639	Lon11	0.33	3.45 E-07	0.60	–
15	23,500,595	rs110052834	Lon12	0.39	1.87 E-07	0.46	NCAM1
16	13,142,992	rs136880011	PL	0.14	7.51 E-08	0.55	RGS18
17	39,552,453	rs135929367	Lon11	0.21	4.18 E-08	0.81	RAPGEF2
17	70,229,172	rs135552789	PL	0.48	1.63 E-07	0.52	RNF185, LIMK2, PIK3IP1, PATZ1, DRG1, PISD
18	13,532,413	rs134612709	Lon13, Lon14, Lon15	0.45	1.12 E-08, 1.23 E-09, 1.11 E-07	0.69, 0.73, 0.63	CA5A, BANP
18	62,531,696	rs134066287	Lon14	0.21	1.47 E-07	0.48	RDH13, NCR1, FCAR, KIR2DL5A, KIR3DL1, KIR2DS1, KIR3DL2
20	30,741,701	rs41567172	ML	0.15	4.07 E-07	0.43	FGF10
22	19,689,530	rs136814811	PL	0.50	3.85 E-07	0.54	GRM7
23	40,794,975	rs109782010	Lon12	0.37	4.58 E-11	1.01	LOC574091, GMPR, MYLIP
24	4,941,502	rs109618980	HL, Lon14	0.25	1.28 E-07, 2.40 E-08	0.51, 0.59	NETO1
24	39,596,108	rs110544386	PL	0.22	1.39 E-08	0.58	–
24	50,494,672	rs110534364	HL	0.10	1.27 E-09	0.66	MRO, ME2, ELAC1, SMAD4
24	52,840,962	rs136448037	Lon12, Lon15	0.37	1.19 E-10, 3.27 E-08	0.80, 0.59	–
25	3,628,922	rs109064423	PL	0.39	8.83 E-08	0.57	TFAP4, GLIS2, CORO7, VASN, DNAJA3, NMRAL1, HMOX2, CDIP1, UBALD1, MGRN1, NUDT16L1, ANKS3, C25H16orf71
25	28,648,581	rs109457044	Lon15	0.16	9.62 E-08	0.62	TYW1, CALN1
26	1,207,558	rs109520495	Lon12	0.15	2.30 E-07	0.51	–
27	42,337,167	rs110187242	Lon11	0.42	1.34 E-07	0.48	UBE2E1, UBE2E2, MIR6532
28	22,970,021	rs42105434	Lon11, Lon12	0.05	4.97 E-08, 3.83 E-07	0.63, 0.50	CTNNA3
X	7,058,580	rs134612804	HL	0.16	6.92 E-09	0.67	
X	7,387,994	rs135876977	Lon11	0.30	2.58 E-12	1.43	GRIA3
X	7,811,883	rs41622390	Lon13, Lon14	0.08	4.39 E-09, 2.18 E-07	0.64, 0.49	GRIA3, THOC2
X	32,975,187	rs137272895	Lon13	0.41	6.21 E-09	0.96	–
X	46,704,483	rs137840659	Lon13, Lon14	0.40	5.41 E-08, 8.61 E-09	0.59, 0.62	–
X	55,267,455	rs137389486	PL	0.40	3.89 E-09	0.85	RNF128
X	52,406,362	rs137119176	HL, ML, Lon13	0.25	1.47 E-08, 4.37 E-08, 9.37 E-10	0.81, 0.99,0.75	TCEAL1, MORF4L2, GLRA4, PLP1, RAB9B
X	57,778,726	rs41612337	Lon11, Lon12	0.36	8.51 E-09, 9.69 E-08	1.19, 0.76	GUCY2F, NXT2
X	128,252,398	rs110389261	HL	0.42	1.26 E-07	0.70	PIR, VEGFD, ASB11, ASB9

*^1^The base pair position on chromosome.^2^Lon11, the days from the first calving to the end of the first lactation or culling; Lon12, the days from the first calving to the end of the second lactation or culling; Lon13, the days from the first calving to the end of the third lactation or culling; Lon14, the days from the first calving to the end of the fourth lactation or culling; Lon15, the days from the first calving to the end of the fifth lactation or culling; PL, productive life referring the days from the first calving to culling or death; ML, milking life referring the days from the first calving to culling or death but excluding all dry periods; HL, herd life referring the days from birth to culling or death.^3^Minor allele frequency of the significant SNP.^4^The percentage of the phenotypic variance explained by the significant SNP.^5^Genes harbor or closest (200 kb) to the significant SNPs.*

The SNPs most significantly associated with Lon11, Lon12, Lon13, Lon14, Lon15, HL, PL, and ML were rs135876977, rs109782010, rs41622390, rs134612709, rs134672623, rs135565406, rs109520811, and rs42867525, respectively, in which rs135565406 was most significantly SNP associated with the full lifespan traits and rs135876977 was on one with the partial lifespan traits. Among the significant SNPs, the proportion of phenotypic variance explained by the top SNPs ranged from 0.73% (rs134612709 for Lon15) to 1.43% (rs135876977 for Lon11).

**FIGURE 1:**
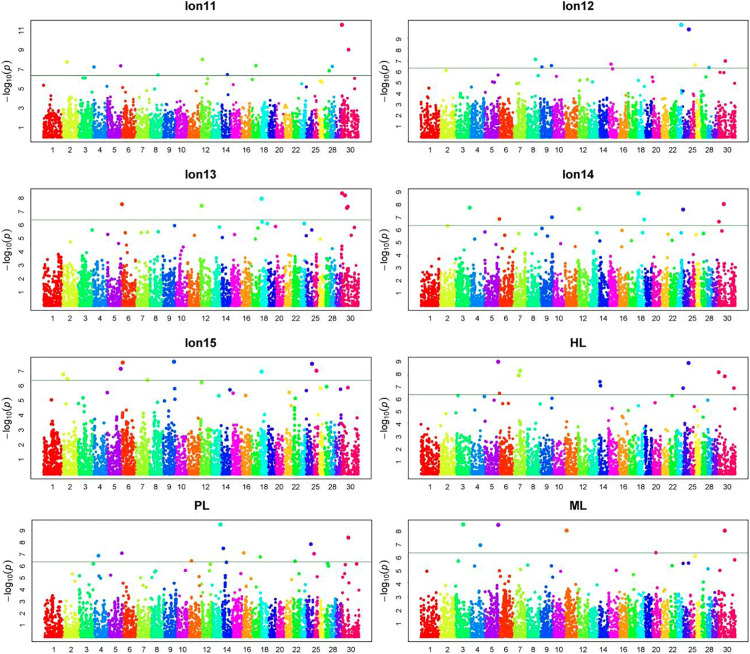
Manhattan plots for 8 longevity traits in Chinese Holsteins. Lon11, the days from the first calving to the end of the first lactation or culling; Lon12, the days from the first calving to the end of the second lactation or culling; Lon13, the days from the first calving to the end of the third lactation or culling; Lon14, the days from the first calving to the end of the fourth lactation or culling; Lon15, the days from the first calving to the end of the fifth lactation or culling; PL, productive life referring the days from the first calving to culling or death; ML, milking life referring the days from the first calving to culling or death but excluding all dry periods; HL, herd life referring the days from birth to culling or death.

**FIGURE 2:**
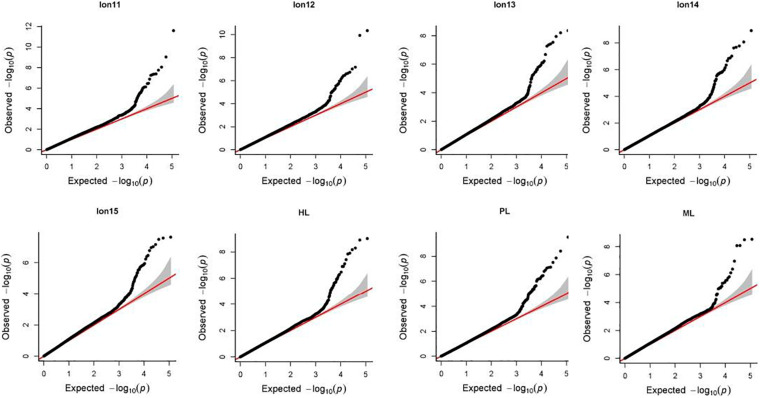
Quantile-quantile (Q-Q) plots of genome-wide association study for 8 longevity traits in Chinese Holsteins. Lon11, the days from the first calving to the end of the first lactation or culling; Lon12, the days from the first calving to the end of the second lactation or culling; Lon13, the days from the first calving to the end of the third lactation or culling; Lon14, the days from the first calving to the end of the fourth lactation or culling; Lon15, the days from the first calving to the end of the fifth lactation or culling; PL, productive life referring the days from the first calving to culling or death; ML, milking life referring the days from the first calving to culling or death but excluding all dry periods; HL, herd life referring the days from birth to culling or death.

Manhattan plots and Q-Q plots for each longevity trait in Chinese Holsteins are presented in Figures 1, 2. The inflation factors λ (Supplementary Table 1) ranged from 1.06 for PL to 1.15 for Lon11. Results from the Q-Q plots and λ showed that the population stratification was well controlled, since the deviation of the observed distribution of the P-values from the expected distribution was minor.

### Annotation of Candidate Genes

Genes harboring or closest (with 200 kb distance) to the significant SNPs were suggested as potential candidate genes for longevity traits. By using this strategy, a total of 106 protein-coding genes and 1 micro RNAs were identified as candidate genes for longevity traits in Chinese Holsteins (Table 4). There are 13 significant SNPs located within CACNA2D1, NAF1, SRD5A2, CDADC1, NCAM1, CAR, HMOX2, CALN1, UBE2E2, CTNNA3, GRIA3, and GUCY2F genes. Furthermore, genes NEFM (rs43559099 and rs134248248), NEFL (rs43559099 and rs134248248), and GRIA3 (rs135876977and rs41622390) harbor significant SNPs associated with more than one longevity trait. In terms of chromosomes, there were the most candidate genes in Chromosome X (14 genes), BTA25 (15 genes), BTA 7 (14 genes), and BTA 18 (9 genes). We listed all of the candidate genes detected in current study together with their functions and associated traits information in Supplementary Table 2, including their association with important economic or functional traits in dairy cattle, beef cattle, human, sheep, goat, laying hens, and pig. The most relevant genes for longevity traits were introduced in discussion.

## Discussion

### Longevity in Chinese Holsteins

Our study showed that the average number of lactations in Chinese Holsteins was 2.7, based on the data of dairy cows born from 1999 to 2017. A previous study using data from 1990s showed that Chinese Holsteins usually culled or dead at 3.4 - 4.0 lactations (Chu et al., 1995; Li et al., 2000), which was 20.5 - 32.5% higher than that observed in current study. There are many possible reasons for the shorter longevity in current Chinese Holstein population. The downtrend of genetic merit on longevity trait in Chinese Holstein population is important reason. On one hand, no direct selection for longevity has been performed in Chinese Holsteins since longevity has not yet been included in the selection index due to the difficulty to collect records of longevity. The longevity has not received the attention it deserves in the past, and a national data collecting system has not yet been established. On the other hand, the intensive selection for milk production traits, which had negative genetic correlations with the longevity traits, has been performed in Chinese Holsteins since the 1990s. Because of its huge impact on the profit of dairy farm, the longevity in dairy cows should not be neglected in the breeding scheme. A similar trend of decrease in the number of lactations was also observed in the United States Holstein population, dating back to the 1990s (Hare et al., 2006). Interestingly, by including the longevity (productive life) into the selection index (TPI), a slight rise of longevity has been achieved since 1994 (Garcia-Ruiz et al., 2016). The example from the United States Holsteins showed that the genetic improvement of longevity can be achieved by selection, when proper longevity traits are employed with sufficient selection intensity. Actually, longevity trait was gradually included into selection index in many countries since 1990s, such as Germany (RZG), Canada (LPI), Denmark (NTM), Netherlands (NVI), and Australia (BPI). The weight of longevity in total selection index ranged from 5 to 20% in various country.

### Heritabilities and Correlations

In the present study, the estimated heritabilities of three full lifespan traits were relatively low (0.038 ∼ 0.040), which was within the range (0.01 ∼ 0.10, Sasaki, 2013) of a previous study in United States and Canadian Holsteins for herd life, milking life, total productive life, and the number of lactation. In the present study, high genetic correlations (0.994 ∼ 0.998) among herd life, total productive life and milking life were found, which was consistent with the previous findings that the genetic correlations among full lifespan traits are generally higher than 0.90 (Klassen et al., 1992; Short and Lawlor, 1992; Chauhan et al., 1993; Jairath et al., 1994). For example, there were a high genetic correlation (0.986) between milking life and total productive life in Dutch Holsteins (Vollema and Groen, 1996) and high genetic correlations (0.98 ∼ 1.00) among milking life, total productive life and number of lactation in Canadian Holsteins (Jairath et al., 1994). Because of the high genetic correlations, only one of three full lifespan traits is needed in the breeding program in order to select for longevity representing the full lifespan. In the present study, heritabilities for partial productive life ranged from 0.051 to 0.090, which is similar to the findings (0.022 ∼ 0.090) of crossbred Danish dairy cattle (consisting of Danish Holstein, Jersey, and Red cattle) (Clasen et al., 2017). Furthermore, the heritabilities for partial productive life was slightly higher than those of full lifespan traits (0.038 ∼ 0.040) in current study. The genetic parameters for longevity estimated by the current study were reliable; and performing the genetic evaluation on longevity traits was feasible in Chinese Holstein population.

Based on the breeder’s equation ($△\,\text{BV}/\text{t}=\frac{{\text{r}}_{\mathrm{BV},\hat{\text{BV}}}\,\text{i}\,\sigma_{\text{BV}}}{\text{L}}$), the genetic gain (△BV/t) of longevity from direct selecting the full lifespan traits was relatively slow due to the long generation interval (L) to obtain the phenotypes, which makes the partial productive life traits become attractive. For Chinese Holstein cows born in 2005, it needs 4.96 years on average to obtain phenotype records for total productive life of these animals, while it is 4.35 years for Lon13 on average. In current study, the genetic correlations among partial productive life traits ranged from 0.648 between Lon11 and Lon15 to 0.963 betweenLon14 and Lon15, which is similar to the results in Nordic Holsteins from the Nordic Genetic Evaluation^4^. With the increase of lactation number, the information the partial productive life traits carried are closer to the total productive life, and correspondingly, the genetic correlations between total productive life and partial productive life traits gradually increased. The high genetic correlations between the total productive life and the partial productive life indicates the potential to implement an early selection on longevity using the partial productive life. To balance between a high genetic correlation with the total productive life and data availability, Lon13 (from the first calving to the end of third lactation or culling) could be considered as the target trait while keeping other partial productive life traits as information traits (only used in genetic evaluation by multi-trait model to increase prediction for the target, but not included in selection index). Further studied needed to be done in order to confirm the reasoning in using the traits Lon13 for selecting longevity in Chinese Holstein.

### GWAS

In current study, two significant SNPs (rs137712544 and rs110628337) on BTA2 are within the reported QTL (37,604,171-49,295,365 bp) for the total productive life in German Holsteins (Kuhn et al., 2003). The gene RPRM within this QTL was considered as a candidate gene for longevity, which is a pleiotropic gene involved in suppression of cancer, regulation of mitotic cell cycle, cell cycle arrest, and regulation of survival. The motif prediction and comparison analysis of protein structure for the gene RPRM showed that it plays important roles on bovine fertility, including sexual maturation, steroidogenesis, gametogenesis, gonadal differentiation and gonadotrophin secretion (Durosaro et al., 2015). In the present study, the SNP rs41622390 located within the gene GRIA3 was significant associated with Lon13 and Lon14, and it also was a top associated SNP (P = 4.39E-09) for Lon13. In a study on United States Holsteins (Cole et al., 2011), the gene GRIA3 was reported to be associated with total productive life, somatic cell score, and daughter pregnancy rate. Result from current study confirmed the findings in United States Holsteins, and the gene GRIA3 was considered as a candidate gene for longevity in Holsteins.

The genes GTF2H5 and CA5A are associated with more than one longevity traits and being detected by the top associated SNP for Lon15 (rs134672623) and Lon14 (rs134612709), respectively. The gene GTF2H5, participating in the interstrand adducts removal process of DNA repair, was reported to be associated with mastitis (Chen et al., 2015) and lipomatous myopathy (Peletto et al., 2017) in cattle, lentivirus susceptibility in sheep (White et al., 2012), ovarian cancer (Gayarre et al., 2016), and trichothiodystrophy (Michalska et al., 2019) in human. The gene CA5A may play an important role in ureagenesis and gluconeogenesis and participates in a variety of biological processes, including respiration, calcification, acid-base balance, bone resorption, and the formation of aqueous humor, cerebrospinal fluid, saliva, and gastric acid (van Karnebeek et al., 2014). This gene was reported to be associated with heat stress in African indigenous cattle (Taye et al., 2017), somatic cell count in Chinese dairy cow (Chen et al., 2015) and European Holsteins (Wijga et al., 2011), and productivity and environmental adaptation traits in Rustaqi and Jenoubi cattle (Iraqi indigenous cattle) (Alshawi et al., 2019). Therefore, GTF2H5 and CA5A were suggested as the candidate genes for longevity in Holsteins. Furthermore, the genes RIBC2, FBLN1, and ATXN10 (identified by the association with HL, ML, PL, Lon11 and Lon15), NPY1R and NAF1 (identified by association with HL, Lon13, and Lon14), and TCEAL1, MORF4L2, GLRA4, PLP1, and RAB9B (identified by association with HL, ML, and Lon13) are novel findings from current study. Beside the genes FBLN1, NPY1R, and MORF4L2 associated with milk production, fertility and health traits in previous study (Supplementary Table 2), few literatures reported that these novel genes are associated with longevity or related traits. These genes could be potential candidate genes for longevity.

Among all 55 significant SNPs identified by the present study, most of them were within the reported QTL for calving traits (26 SNPs, mainly including calving ease, stillbirth, calf size, and birth weight), health traits (24 SNPs, mainly including mastitis, somatic cell count/score, abomasum displacement and ketosis), fertility traits (22 SNPs, mainly including non-return rate and gestation length), and immunity (16 SNPs, mainly including blood immunoglobulin G level). This phenomenon has also been observed in the previous GWAS for longevity in North American (Nayeri et al., 2017), United States (Cole et al., 2011), and Nordic (Zhang et al., 2016). Holstein population, Nordic Red cattle population (Zhang et al., 2016), and German-Austrian Fleckvieh population (Mészáros et al., 2014), where the most significant SNPs were located within the previously identified QTL regions for production, type, diseases resistance, somatic cell count/score, fertility and calving traits. All longevity traits with different definitions essentially measure the resistance to culling caused by various problems, such as low productivity, reproduction disorders, and health problems. In the Chinese Holstein population, reproduction disorders (e.g., dystocia and infertility, accounting for 19%) and udder health problems (e.g., mastitis, accounting for 9%) were the main culling causes, according the survey data in the year Yan et al. (2016). The finding of shared genetic markers between longevity and other functional traits further confirms the fact that longevity is genetically related to health, fertility and calving traits. Among 109 potential candidate genes for longevity detected in the present study, 63 genes had been reported to be associated with clinical mastitis, somatic cell count/score, fertility traits (the first calving age, pregnancy rate and the age at puberty), calving traits (birth weight and calving ease score), other health traits (tick resistance, ketosis, brucellosis, and foot-and-mouth disease), embryonic development and heat stress in cattle. For example, the gene CACNA2D1 encodes a member of the alpha-2/delta subunit family, which is associated with voltage-gated calcium channels. In Indian Sahiwal cattle (Magotra et al., 2019), and Chinese Holstein, Sanhe, and Simmental cattle (Deng et al., 2011; Yuan et al., 2011a, b), the gene CACNA2D1 were significantly associated with somatic cell score or clinical mastitis. The gene FGF10 are involved in various cellular processes, including chemotaxis, cell migration, differentiation, survival, apoptosis, embryonic development and angiogenesis, which can inhibit dominant follicle growth and estradiol secretion (Gasperin et al., 2012), and plays important roles follicle selection (Diogenes et al., 2017) and vitro embryo production (Castilho et al., 2017) in cattle. The gene DNAJA3 plays an important antiviral role against foot-and-mouth disease by both degrading VP1 and restoring of IFN-β signaling pathway (Zhang et al., 2019). Furthermore, in post-GWAS analysis for mastitis resistance (Cai et al., 2018) and transcriptome comparative analysis for brucellosis (Rossetti et al., 2011) in cattle, the gene DNAJA3 also showed the significant statistical signal. We suggested that the genes CACNA2D1, FGF10 and DNAJA3 can be considered as candidate genes for longevity.

In current study, the X chromosome had the greatest number of significant SNPs (Table 4), which was in agreement with GWAS results for total productive life trait in United States Holsteins (Cole et al., 2011). In the present study, many genes close to the significant SNPs on X chromosome were found, including THOC2, RNF128, TCEAL1, MORF4L2, PLP1, RAB9B, GUCY2F, NXT2, PIR, VEGFD, ASB11 and ASB9. Excluded the gene THOC2 (associated with fertility in bovine), MORF4L2 (associated with heat stress in Holstein), VEGFD and ASB11 (associated with fertility in pig), no studies about the functions of these genes on various traits in livestock (especially in cattle) were available (Supplementary Table 2).

## Conclusion

This is the first study to investigate the genetic architecture of longevity traits representing a full or a partial lifespan simultaneously. Because of high genetic correlations with the total productive lifespan traits and higher heritability, the partial productive life measured as days from the first calving to the end of third lactation or culling (Lon13) could be a good alternative trait for early selection on longevity. The shared underlying biological processes among different longevity traits were further confirmed by the detection of shared significant variants. The genes RPRM, GRIA3, GTF2H5, CA5A, CACNA2D1, FGF10, and DNAJA3 were suggested to be candidate genes for longevity in Holsteins, which could be used to pinpoint causative mutations and further benefit genomic prediction for longevity in dairy cattle. This study proved the feasibility of genetic evaluation on longevity in Chinese dairy population, and it should be considered in selection index of Chinese dairy cattle.

## Methods

### Data

#### Phenotype and Pedigree

The dates of birth, calving, drying, and culling or death were collected for 132,690 Chinese Holstein cows born from 1999 to 2017, which were raised in 31 herds in China, including 22 herds in Beijing, two herds in Hebei, and one herd each in Tianjin, Yunnan, Henan, Heilongjiang, Jilin, and Inner Mongolia, respectively. These electronic records of each cow were extracted from the farm management software (AfiFarm^5^). A total of eight longevity traits were analyzed in this study, including three traits representing full lifespan and five traits representing partial lifespan. Traits representing full lifespan were herd life (HL) referring the days from birth to culling or death, total productive life (PL) referring the days from the first calving to culling or death, and milking life (ML) referring the days from birth to culling or death but excluding all dry periods. Traits representing partial lifespan were the days of a cow staying during the period from the first calving to the end of the first (Lon11), second (Lon12), third (Lon13), fourth (Lon14), or fifth lactation (Lon15), according to the study by Clasen et al. (2017). In order to reflect the real longevity of cows, no correction for production or for functional performance has been performed for any of the eight longevity traits. Only cows with age at first calving ranged from 600 to 1800 days and did not change herds during the data collection period were kept for further analyses. Besides, for HL, PL and ML, only culled or dead cows were kept; whereas for all five partial lifespan traits, only cows culled or finished the corresponding lactation were kept. Ultimately, there were 78,227 cows available for HL, PL, and ML, and 103,479, 90,279, 82,826, 79,571, and 78,144 cows available for Lon11, Lon12, Lon13, Lon14, and Lon15, respectively. To obtain an adequate pedigree, cows with phenotypic records were traced back as many generations as possible. The final pedigree included 437,418 females and 12,401 males born from 1969 to 2017.

#### Genotype

A total of 2,629 cows born from 2003 to 2015 were genotyped with the Illumina 150 K bovine bead chip (Illumina, Inc., San Diego, CA, United States). Genotype imputation was performed using the Beagle 5.1 software (Browning and Browning, 2009). The SNPs were removed from the dataset if they exhibited: (1) Minor allele frequency (MAF) lower than 0.05; (2) Fisher’s exact test P-value for Hardy-Weinberg Equilibrium (HWE) less than 10-6; or (3) unknown position. Quality control for animals available in each trait was performed, respectively. Ultimately, the numbers of SNPs used for GWAS ranged from 116,547 for PL to 116,570 for Lon12 (Supplementary Table 3).

### Statistical Modeling

#### Genetic Parameter Estimation

Variance and co-variance components for eight longevity traits were estimated using the average information restricted maximum likelihood algorithm implemented in the DMU software (Madsen et al., 2006). Heritabilities were estimated using single-trait animal models. Correlations between the longevity traits within the same group were estimated using a multi-trait model, that is, a three-trait animal model for the three traits representing full lifespan (HL, PL, and ML), and a two-trait animal model for each pair of the five partial lifespan traits (Lon11, Lon12, Lon13, Lon14, or Lon15), instead of a five-trait animal model which did not meet the convergence criteria. Genetic correlation between PL and any one trait measuring partial productive life was estimated using a two-trait animal model. The effects included in the model were the same for all longevity traits:

y=a⁢f⁢c+h⁢y+y⁢s+a+e

where y is the vector of phenotypes for longevity traits, afc is the vector of fixed effect of age at first calving (≤22, 23, 24, 25, 26, 27, 28, 29, and ≥30 month); hy is a vector of fixed effect of herd-birth year; ys is a vector of fixed effect of birth year-season; a is the vector of additive genetic effects; and e is the vector of random residual effects. It was assumed that a ∼$N\,\left(0,A\,\sigma_{\text{a}}^{2}\right)$ and e ∼$N\,\left(0,I\,\sigma_{\text{e}}^{2}\right),$ where A is the matrix of additive genetic relationships constructed from the pedigree, $\sigma_{\text{a}}^{2}$ is the additive genetic variance, I is the identity matrix, and $\sigma_{\text{e}}^{2}$ is the residual variance.

### Genome-Wide Association Studies

The de-regressed estimate breeding values (dEBV) (VanRaden et al., 2009) generated from BLUP solutions of the above mentioned single-trait model were used as pseudo phenotypes for GWAS. The descriptive statistics of dEBV for 8 longevity traits are listed in Supplementary Table 4. A Fixed and Random Circuitous Probability Unification model implemented in the FarmCPU software (Liu et al., 2016) was used to perform single-trait GWAS. To reduce false positives caused by the population structure, top 50 or 90 principal components (PCs) calculated by the PLINK software^6^ were added to the GWAS model as covariates to control the inflation factor λ below 1.2. The proportions of phenotypic variance explained by these PCs are listed in Supplemental Table 3. Bonferroni correction was used to control the false positive resulted from multiple comparisons. The significance threshold was defined as 0.05/N, where N was the number of SNPs being tested. The Quantile-quantile (Q-Q) plots and the genomic inflation factor λ (Devlin and Roeder, 1999) were used to determine whether the observed distributions of --log (P-value) was against the expected distribution under no association hypothesis. The ARS_UCD1.2 assembly from the UC Santa Cruz genome annotation database^7^ was used to refer the SNP positions and search the genes related to the significant SNPs.

## Data Availability Statement

The data analyzed in this study is subject to the following licenses/restrictions: This manuscript utilizes proprietary data. Requests to access these datasets should be directed to YW, wangyachun@cau.edu.cn.

## Ethics Statement

Ethical review and approval was not required for the animal study because All phenotypic data were recorded as part of routine dairy cattle management and genetic evaluations. The DNA samples were obtained for the purpose of routine genomic evaluations in previous projects. Thus, no additional animal handling or experiment was performed specifically for this study.

## Author Contributions

HZ, GS, and YW organized the study. HZ and AL led the manuscript preparation. HZ performed the data analysis and HL did the data curation of genotype. XY and XL did the data curation of phenotype. LL provided support for collection of raw data. All authors contributed to the article and approved the submitted version.

## Conflict of Interest

The authors declare that the research was conducted in the absence of any commercial or financial relationships that could be construed as a potential conflict of interest.

## Footnotes

1 http://www.interbull.org/index

2 https://www.nordicebv.info/

3 https://www.dac.com.cn/

4 https://www.nordicebv.info/

5 http://www.afimilk.com.cn

6 https://www.cog-genomics.org/plink2/

7 https://genome.ucsc.edu

## Supplementary Material

Supplementary Figure 1 Distribution of eight longevity traits in Chinese Holsteins.

Supplementary Table 1 The number of principal components (PC) added in GWAS model, the proportion of dEBV variance explained by these principal components and the inflation factors (λ) of single-trait GWAS for each longevity trait.

Supplementary Table 2 The potential candidate genes related to longevity and their functions.

Supplementary Table 3 Number of SNPs and genotyped animals kept for association analysis on each longevity trait in Chinese Holstein cattle.

Supplementary Table 4 Descriptive statistics of de-regressed estimate breeding values for longevity traits in Chinese Holsteins.

## Abbreviations

GWAS: genome-wide association study
SNP: single nucleotide polymorphism
dEBV: de-regressed estimate breeding value
PC: principal component
QTL: quantitative trait locus
MAF: minor allele frequency
Chr: chromosome
bp: base pair
Lon11: the days from the first calving to the end of the first lactation or culling
Lon12: the days from the first calving to the end of the second lactation or culling
Lon13: the days from the first calving to the end of the third lactation or culling
Lon14: the days from the first calving to the end of the fourth lactation or culling
Lon15: the days from the first calving to the end of the fifth lactation or culling
PL: productive life referring the days from the first calving to culling or dead
ML: milking life referring the days from the first calving to culling or death but excludes all dry periods
HL: herd life referring the days from birth to culling or death
SCC: somatic cell count
SCS: somatic cell score.
