# Supplementary material for: Genetic Parameters and Genome-Wide Association Studies of Eight Longevity Traits Representing Either Full or Partial Lifespan in Chinese Holsteins
Source: Front Genet. 2021 Feb 25;12:634986. doi: 10.3389/fgene.2021.634986 (PMC7947242; doi:10.3389/fgene.2021.634986)
Supplement: Supplementary Figure 1 — Distribution of eight longevity traits in Chinese Holsteins. [file Data_Sheet_1.docx]

**SUPPLEMENTARY MATERIAL 1**


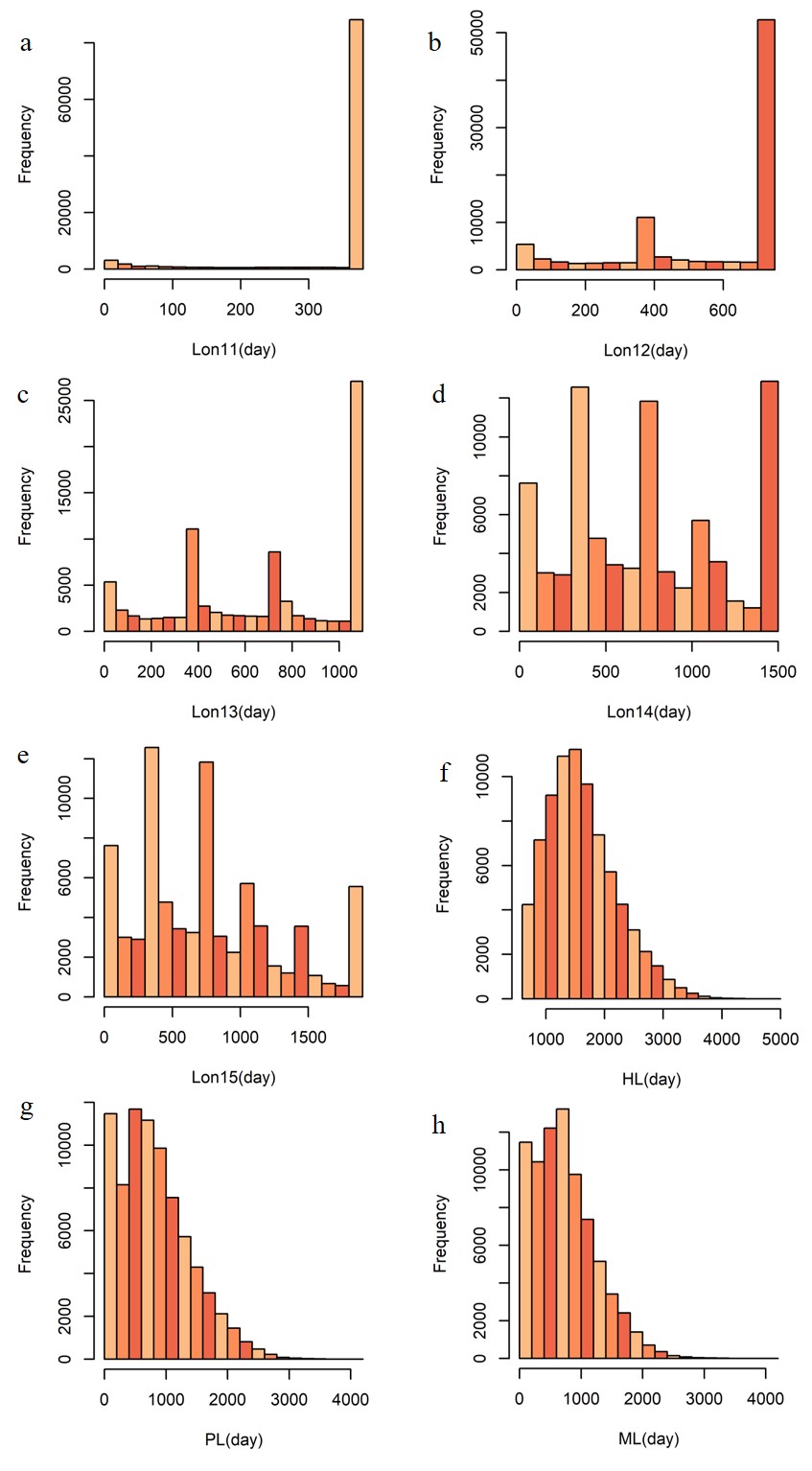


Supplementary Figure 1. Histogram of eight longevity traits in Chinese Holsteins^1^

^1^Lon11, the days from the first calving to the end of the first lactation or culling; Lon12, the days from the first calving to the end of the second lactation or culling; Lon13, the days from the first calving to the end of the third lactation or culling; Lon14, the days from the first calving to the end of the fourth lactation or culling; Lon15, the days from the first calving to the end of the fifth lactation or culling; PL, productive life referring the days from the first calving to culling or dead; ML, milking life referring the days from the first calving to culling or death but excludes all dry periods; HL, herd life referring the days from birth to culling or death.

Supplementary Table 1. The number of principal components (PC) added in GWAS model, the proportion of dEBV variance explained by these principal components and the inflation factors (λ) of single-trait GWAS for each longevity trait

| Traits^1^ | PC | The explained of phenotypic variance (%) | λ |
| --- | --- | --- | --- |
| HL | PC50 | 0.2540 | 1.11 |
| PL | - | - | 1.06 |
| ML | PC90 | 0.3261 | 1.13 |
| Lon11 | PC90 | 0.3290 | 1.15 |
| Lon12 | PC90 | 0.3289 | 1.11 |
| Lon13 | PC50 | 0.2568 | 1.08 |
| Lon14 | PC50 | 0.2569 | 1.08 |
| Lon15 | PC90 | 0.3293 | 1.11 |

^1^Lon11, the days from the first calving to the end of the first lactation or culling; Lon12, the days from the first calving to the end of the second lactation or culling; Lon13, the days from the first calving to the end of the third lactation or culling; Lon14, the days from the first calving to the end of the fourth lactation or culling; Lon15, the days from the first calving to the end of the fifth lactation or culling; PL, productive life referring the days from the first calving to culling or dead; ML, milking life referring the days from the first calving to culling or death but excludes all dry periods; HL, herd life referring the days from birth to culling or death.
