# Supplementary material for: Genetic Parameters and Genome-Wide Association Studies of Eight Longevity Traits Representing Either Full or Partial Lifespan in Chinese Holsteins
Source: Front Genet. 2021 Feb 25;12:634986. doi: 10.3389/fgene.2021.634986 (PMC7947242; doi:10.3389/fgene.2021.634986)
Supplement: Supplementary Table 2 — The potential candidate genes related to longevity and their functions. [file Data_Sheet_2.docx]

**SUPPLEMENTARY MATERIAL 2**

Supplementary Table 2. The potential candidate genes related to longevity and their functions

| Genes | Gene full name | Functions^1^ | Associated traits^2^ | |
| --- | --- | --- | --- | --- |
| *CALCRL* | calcitonin receptor like receptor | - | first calving age[1] | |
| *RPRM* | reprimo, TP53 dependent G2 arrest mediator homolog | sexual maturation, steroidogenesis, gametogenesis, gonadal differentiation and gonadotrophin secretion[2] | fertility[2] | |
| *TFPI2* | tissue factor pathway inhibitor 2 | proliferation and death of smooth muscle cells [3] | somatic cell score [3] | |
| *GNGT1* | G protein subunit gamma transducin 1 | control of cell migration and adhesion [3] | somatic cell score [3] | |
| *GNG11* | G protein subunit gamma 11 | control of cell migration and adhesion [3] | somatic cell score [3] | |
| *BET1* | Bet1 golgi vesicular membrane trafficking protein | - | - | |
| *CACNA2D1* | calcium voltage-gated channel auxiliary subunit alpha2delta 1 | associated with voltage-gated calcium channels [4] | clinical mastitis [4, 5], somatic cell score [4, 6, 7], carcass trait [8], meat quality [8], milk yield [5] | |
| *ING3* | inhibitor of growth family member 3 | melanoma proliferation[9] | pig mentation traits[9] | |
| *RIBC2* | RIB43A domain with coiled-coils 2 | differential methylation in bovine fetus development[10] | - | |
| *FBLN1* | fibulin 1 | development and cell differentiation of mammary glands[11] | milk protein percent, milk protein yield[12] | |
| *ATXN10* | ataxin 10 | - | - | |
| *NPY1R* | neuropeptide Y receptor Y1 | physiological control of energy homeostasis [13] | fertility[14], maternal behavior[15] | |
| *NAF1* | nuclear assembly factor 1 ribonucleoprotein | cellular TERT activity, cancer and aging[16] | human disease[16] | |
| *PRSS12* | serine protease 12 | - | - | |
| *NDST3* | N-deacetylase and N-sulfotransferase 3 | heparan sulfate and heparin metabo-lism [17] | - | |
| *CIRBP* | cold inducible RNA binding protein | cell stress response [18] | - | |
| *FAM174C* | family with sequence similarity 174 member C | - | - | |
| *PWWP3A* | PWWP domain containing 3A, DNA repair factor | - | - | |
| *NDUFS7* | NADH: ubiquinone oxidoreductase core subunit S7 | - | - | |
| *GAMT* | guanidinoacetate N-methyltransferase | energy, creatine biosynthesis [19] | tenderness[19] | |
| *DAZAP1* | DAZ associated protein 1 | cellular activities, cell proliferation[20] | semen traits[21] | |
| *RPS15* | ribosomal protein S15 | ribosomal protein assembly and translational regulation [22]， | clinical mastitis[23, 24] | |
| *C7H19orf25* | chromosome 7 C19orf25 homolog | - | somatic cell score [25] | |
| *REEP6* | receptor accessory protein 6 | embryonic development, sperm motility and structure, regulation of cell differentiation and proliferation, and bulk protein degradation [26] | somatic cell score[25], horse fertility[26] | |
| *MBD3* | methyl-CpG binding domain protein 3 | early embryonic development and pluripotency [27] | fertility[27] | |
| *UQCR11* | [ubiquinol-cytochrome c reductase, complex III subunit XI](https://www.ncbi.nlm.nih.gov/gene/281570) | subcutaneous adipose tissue[28], mitochondrial energy production functions[29] | - | |
| *TCF3* | transcription factor 3 | - | daughter pregnancy rate[30] | |
| *NR3C1* | [nuclear receptor subfamily 3 group C member 1](https://www.ncbi.nlm.nih.gov/gene/281946) | glucocorticoid receptor, stress response[31] | meat quality[31], fertility[32] | |
| *PCSK1* | [proprotein convertase subtilisin/kexin type 1](https://www.ncbi.nlm.nih.gov/gene/281967) | central and peripheral energy metabolism [33] | growth, birth weight[34, 35] | |
| *NEFM* | [neurofilament medium](https://www.ncbi.nlm.nih.gov/gene/281347) | - | - | |
| *NEFL* | [neurofilament light](https://www.ncbi.nlm.nih.gov/gene/281348) | - | fertility[36] | |
| *FILIP1* | [filamin A interacting protein 1](https://www.ncbi.nlm.nih.gov/gene/514193) | - | - | |
| *SENP6* | [SUMO specific peptidase 6](https://www.ncbi.nlm.nih.gov/gene/533853) | muscle lipid composition [37] | age at puberty[36] | |
| *SERAC1* | [serine active site containing 1](https://www.ncbi.nlm.nih.gov/gene/519860) | - | mouse sterility[38], marbling score[39] | |
| *GTF2H5* | general transcription factor IIH subunit 5 | - | health trait[40], mastitis[41], ovine lentivirus susceptibility[42] | |
| *DYNLT1* | [dynein light chain Tctex-type 1](https://www.ncbi.nlm.nih.gov/gene/282380) | - | health trait[40], backfat thickness[43] | |
| *QKI* | [QKI, KH domain containing RNA binding](https://www.ncbi.nlm.nih.gov/gene/493722) | glial cell differentiation[44] | feed efficiency[45] | |
| *XDH* | xanthine dehydrogenase | synthesis of reactive oxygen and reactive nitrogen species[46] | ticks resistance[45], clinical mastitis[47] | |
| *MEMO1* | mediator of cell motility 1 | - | heifer pregnancy[48] | |
| *SRD5A2* | steroid 5 alpha-reductase 2 | Metabolism of lipids and lipoproteins[49] | ketosis[49], fertility[50], goat fertility[51], human semen traits[52] | |
| *PKDCC* | protein kinase domain containing, cytoplasmic | - | heat stress[53], growth trait[151] | |
| *EML4* | EMAP like 4 | - | - | |
| *COX7A2L* | cytochrome c oxidase subunit 7A2 like | promotes respiratory supercomplex assembly and regulates energy generation[54, 55] | fertility[54, 56], feed efficiency[55] | |
| *FNDC3A* | fibronectin type III domain containing 3A | - | estrous behavior[57], production traits[58] | |
| *CDADC1* | cytidine and dCMP deaminase domain containing 1 | - | ticks resistance[59], egg weight[60] | |
| *SETDB2* | SET domain bifurcated histone lysine methyltransferase 2 | - | embryonic development[61], goat litter size trait[62] | |
| *PHF11* | PHD finger protein 11 | Immune response[63] | - | |
| *NCAM1* | neural cell adhesion molecule 1 | - | health traits[64] | |
| *RGS18* | regulator of G protein signaling 18 | highly expressed in megakaryocytes and platelets[65] | - | |
| *RAPGEF2* | Rap guanine nucleotide exchange factor 2 | - | fertility[14], human gestational age[66] | |
| *RNF185* | ring finger protein 185 | selective mitochondrial autophagy in cultured cells[67] | health traits[68] | |
| *LIMK2* | LIM domain kinase 2 | - | - | |
| *PIK3IP1* | phosphoinositide-3-kinase interacting protein 1 | fatty acid metabolic process[69] | meat quality[69] | |
| *PATZ1* | POZ/BTB and AT hook containing zinc finger 1 | - | health traits[70] | |
| *DRG1* | developmentally regulated GTP binding protein 1 | - | udder texture[71] | |
| *PISD* | phosphatidylserine decarboxylase | - | meat quality, meat production[72] | |
| *CA5A* | carbonic anhydrase 5A | - | heat stress[73], SCC(SCS)[41, 74], productivity and environmental traits[75] | |
| *BANP* | BTG3 associated nuclear protein | - | productivity and environmental traits[75], clinical mastitis[41] | |
| *RDH13* | retinol dehydrogenase 13 | retinoate biosynthesis[76] | health traits[77, 78], feed efficiency[76], milk fat percentage[79] | |
| *KIR2DL5A* | killer cell immunoglobulin-like receptor, two domains, long cytoplasmic tail, 5A | graft-versus-host disease and natural killer cell mediated cytotoxicity[80] | calving performance and body conformation[80], human disease[81] | |
| *FCAR* | Fc fragment of IgA receptor | encoding the immunoglobulin A receptor[82] | - | |
| *NCR1* | natural cytotoxicity triggering receptor 1 | recognition and killing of tumor cells[83] | - | |
| *KIR2DS1* | killer cell immunoglobulin-like receptor, two domains, short cytoplasmic tail, 1 | - | - | |
| *KIR3DL1* | killer cell immunoglobulin-like receptor, three domains, long cytoplasmic tail, 1 | - | - | |
| *KIR3DL2* | killer cell immunoglobulin-like receptor, three domains, long cytoplasmic tail, 2 | - | - | |
| *FGF10* | fibroblast growth factor 10 | developmental of bovine oocytes[84], follicle growth and estradiol secretion[85, 86] | fertility[84-86] | |
| *GRM7* | glutamate metabotropic receptor 7 | - | cold stress[87], production trait[88], human disease[89] | |
| *LOC574091* | nucleoside-diphosphate kinase NBR-A | - | fertility[90][204] | |
| *GMPR* | guanosine monophosphate reductase | the interconversion of purine nucleotides[91] | livestock parasite resistance[92] | |
| *MYLIP* | myosin regulatory light chain interacting protein | - | - | |
| *NETO1* | neuropilin and tolloid like 1 | - | - | |
| *MRO* | maestro | - | - | |
| *ME2* | malic enzyme 2 | - | - | |
| *ELAC1* | elaC ribonuclease Z 1 | - | high altitude adaptation[93] | |
| *SMAD4* | SMAD family member 4 | myostatin signaling[94], the glucose transport[95], bovine preimplantation embryonic development[95] | fertility[95] | |
| *TFAP4* | transcription factor AP-4 | - | clinical mastitis[41], human disease[96] | |
| *GLIS2* | GLIS family zinc finger 2 | - | milk yield[97], clinical mastitis[41] | |
| *CORO7* | coronin 7 | - | - | |
| *VASN* | vasorin | - | clinical mastitis[41, 98] | |
| *DNAJA3* | DnaJ heat shock protein family (Hsp40) member A3 | - | clinical mastitis[98], foot-and-mouth disease[99], brucellosis[100] | |
| *NMRAL1* | [NmrA like redox sensor 1](https://www.ncbi.nlm.nih.gov/gene/534628) | - | - | |
| *HMOX2* | heme oxygenase 2 | - | clinical mastitis[41], fertility[101] | |
| *CDIP1* | cell death inducing p53 target 1 | - | clinical mastitis[98] | |
| *UBALD1* | UBA like domain containing 1 | - | tenderness[102] | |
| *MGRN1* | mahogunin ring finger 1 | - | bull fertility[103],  goat fertility[104], tenderness[102] | |
| *NUDT16L1* | nudix hydrolase 16 like 1 | - | goat fertility[104], sheep fertility and production[105] | |
| *ANKS3* | ankyrin repeat and sterile alpha motif domain containing 3 | - | goat fertility[104] , sheep fertility and production[105], human disease[106] | |
| *C25H16orf71* | chromosome 25 C16orf71 homolog | - | goat fertility[104], | |
| *TYW1* | tRNA-yW synthesizing protein 1 homolog | - | pregnancy rate[103], marbling score[107, 108] | |
| *CALN1* | calneuron 1 | neurological development and behavioural disorders[109] | production[110], feed efficiency[111] | |
| *UBE2E1* | ubiquitin conjugating enzyme E2 E1 | - | - | |
| *UBE2E2* | ubiquitin conjugating enzyme E2 E2 | - | maternal behavior and suckling performance[15], milk fatty acid[112] | |
| *CTNNA3* | catenin alpha 3 | tight junction and the leucocyte transendothelial migration[113] | chest width[114], marbling score[108] | |
| *GRIA3* | glutamate ionotropic receptor AMPA type subunit 3 | - | pregnancy rate, SCS, total productive life[115] | |
| *THOC2* | THO complex 2 | - | fertility[116] | |
| *RNF128* | ring finger protein 128 | - | - | |
| *TCEAL1* | transcription elongation factor A like 1 | - | - | |
| *MORF4L2* | mortality factor 4 like 2 | cell growth and proliferation[117] | embryonic development[117], health trait[118], heat stress[119] | |
| *PLP1* | proteolipid protein 1 | - | - | |
| *GLRA4* |  |  |  | |
| *RAB9B* | RAB9B, member RAS oncogene family | - | - | |
| *GUCY2F* | guanylate cyclase 2F, retinal | - | - | |
| *NXT2* | nuclear transport factor 2 like export factor 2 | - | - | |
| *PIR* | pirin | - | - | |
| *VEGFD* | vascular endothelial growth factor D | - | pig fertility[120] | |
| *ASB11* | ankyrin repeat and SOCS box containing 11 | ubiquitination and proteasomal degradation[121] | pig fertility[122] | |
| *ASB9* | ankyrin repeat and SOCS box containing 9 | ubiquitination and proteasomal degradation[121] | - |  |

Note：1“-” represents no information about the candidate genes; 2 excepted the traits with specific marked species or livestock species, all other associated traits belong to cattle, including Bos Taurus and Bos indicus.

**References:**

1. Utsunomiya ATH, Boison SA, Santos DJAD, Utsunomiya YT, Machado MA, Verneque RS, Sölkner J, Garcia JF, Fonseca RD, Da Silva MVGB: **Genome Wide Scan for Age at First Calving in Gyr Dairy Cattle**. In: *10th World Congress of Genetics Applied to Livestock Production.*; 2014.

2. Durosaro S, Peters S, Adebambo A, Onagbesan O, Sanda A, Olowofeso O, Osho S, Ozoje M: **Computational identification of fertility functions of bovine Reprimo gene**. *Nigerian Journal of Animal Production* 2015, **42**(1):19-29.

3. Wijga S, Bastiaansen JWM, Wall E, Strandberg E, de Haas Y, Giblin L, Bovenhuis H: **Genomic regions associated with somatic cell score in dairy cattle**. In*.* Wageningen: Wageningen Academic Publishers; 2011.

4. Yuan ZR, Li J, Liu L, Zhang LP, Zhang LM, Chen C, Chen XJ, Gao X, Li JY, Chen JB *et al*: **Single nucleotide polymorphism of CACNA2D1 gene and its association with milk somatic cell score in cattle**. *MOL BIOL REP* 2011, **38**(8):5179-5183.

5. Magotra A, Gupta ID, Verma A, Alex R, Mr V, Ahmad T: **Candidate SNP of CACNA2D1 Gene Associated with Clinical Mastitis and Production Traits in Sahiwal (Bos taurus indicus) and Karan Fries (Bos taurus taurus x Bos taurus indicus)**. *ANIM BIOTECHNOL* 2019, **30**(1):75-81.

6. Deng G, Yuan Z, Gao X, Li J, Chen J, Huijiang, Gao, Xu S: **Identification mutation of the CACNA2D1 gene and its effect on somatic cell score in cattle**. *J APPL ANIM RES* 2011, **39**(1):15-18.

7. Yuan Z, Li J, Zhang L, Zhang L, Chen C, Chen X, Gao X, Li J, Chen J, Gao H *et al*: **Novel SNPs polymorphism of bovine CACNA2D1 gene and their association with somatic cell score**. *African Journal of Biotechnology* 2011, **10**(10):1789-1793.

8. Yuan ZR, Xu SZ: **Novel SNPs of the bovine CACNA2D1 gene and their association with carcass and meat quality traits**. *MOL BIOL REP* 2011, **38**(1):365-370.

9. Fan Y, Wang P, Fu W, Dong T, Qi C, Liu L, Guo G, Li C, Cui X, Zhang S *et al*: **Genome-wide association study for pigmentation traits in Chinese Holstein population**. *ANIM GENET* 2014, **45**(5):740-744.

10. Li Y, Tríbulo P, Bakhtiarizadeh MR, Siqueira LG, Ji T, Rivera RM, Hansen PJ: **Conditions of embryo culture from days 5 to 7 of development alter the DNA methylome of the bovine fetus at day 86 of gestation**. *J ASSIST REPROD GEN* 2019:1-10.

11. Farhadian M, Rafat SA, Hasanpur K, Ebrahimie E: **Transcriptome signature of the lactation process, identified by meta-analysis of microarray and RNA-Seq data**. *Journal of Biotechnology Computational Biology and Bionanotechnology* 2018, **99**(2).

12. Raven L, Cocks BG, Pryce JE, Cottrell JJ, Hayes BJ: **Genes of the RNASE5 pathway contain SNP associated with milk production traits in dairy cattle**. *GENET SEL EVOL* 2013, **45**(1):25.

13. Li P, Tiwari HK, Lin WY, Allison DB, Chung WK, Leibel RL, Yi N, Liu N: **Genetic Association Analysis of 30 Genes Related to Obesity in a European American Population**. *INT J OBESITY* 2014, **38**(5):724-729.

14. Neupane M, Geary TW, Kiser JN, Burns GW, Hansen PJ, Spencer TE, Neibergs HL: **Loci and pathways associated with uterine capacity for pregnancy and fertility in beef cattle**. *PLOS ONE* 2017, **12**(12):e188997.

15. Michenet A, Saintilan R, Venot E, Phocas F: **Insights into the genetic variation of maternal behavior and suckling performance of continental beef cows**. *GENET SEL EVOL* 2016, **48**(1):45.

16. Brane A, Tollefsbol T: **Targeting Telomeres and Telomerase: Studies in Aging and Disease Utilizing CRISPR/Cas9 Technology**. *CELLS-BASEL* 2019, **8**(2):186.

17. Jiang Z, Michal JJ, Wu XL, Pan Z, MacNeil MD: **The heparan and heparin metabolism pathway is involved in regulation of fatty acid composition**. *INT J BIOL SCI* 2011, **7**(5):659-663.

18. Bebbere D, Pinna S, Nieddu S, Natan D, Arav A, Ledda S: **Gene expression analysis of ovine prepubertal testicular tissue vitrified with a novel cryodevice (E.Vit)**. *J ASSIST REPROD GEN* 2019, **36**(10):2145-2154.

19. Rosa AF, Moncau CT, Poleti MD, Fonseca LD, Balieiro J, Silva S, Eler JP: **Proteome changes of beef in Nellore cattle with different genotypes for tenderness**. *MEAT SCI* 2018, **138**:1-9.

20. Hou S, Hao Q, Zhu Z, Xu D, Liu W, Lyu L, Li P: **Unraveling proteome changes and potential regulatory proteins of bovine follicular Granulosa cells by mass spectrometry and multi-omics analysis**. *PROTEOME SCI* 2019, **17**:4.

21. Khayatzadeh N, Meszaros G, Utsunomiya YT, Schmitz-Hsu F, Seefried F, Schnyder U, Ferencakovic M, Garcia JF, Curik I, Solkner J: **Genome-wide mapping of the dominance effects based on breed ancestry for semen traits in admixed Swiss Fleckvieh bulls**. *J DAIRY SCI* 2019, **102**(12):11217-11224.

22. LUO XY, HU JC, HOU WR: **cDNA Cloning and Sequences Analysis of RPS15 from the Giant Panda**. *Sichuan Journal of Zoology* 2008, **2**.

23. JATAV P, SODHI M, SHARMA A, MANN S, KISHORE A, SHANDILYA UK, MOHANTY AK, KATARIA RS, YADAV P, VERMA P *et al*: **Identification of internal control genes in milk-derived mammary epithelial cells during lactation cycle of Indian zebu cow**. *ANIM SCI J* 2016, **87**(3):344-353.

24. Gurao A, Kataria R, Singh R, Kashyap RD, Mishra K, Gurao A: **Association analysis of differential expression of beta defensins with mastitis in indicine dairy cattle**. *Indian J Dairy Sci* 2018, **71**(5):534-537.

25. Duran AM, Roman PS, Ruiz LF, Gonzalez PE, Vasquez PC, Bagnato A, Strillacci MG: **Genome-wide association study for milk somatic cell score in holstein cattle using copy number variation as markers**. *J ANIM BREED GENET* 2017, **134**(1):49-59.

26. Stefaniuk M, Ropka-Molik K: **RNA sequencing as a powerful tool in searching for genes influencing health and performance traits of horses**. *J APPL GENET* 2016, **57**(2):199-206.

27. Wilson BC: **MicroRNA-mediated silencing of bovine NANOG and MBD3**.: The University of Waikato; 2015.

28. English AM, Waters SM, Cormican P, Byrne CJ, Fair S, Kenny DA: **Effect of early calf-hood nutrition on the transcriptomic profile of subcutaneous adipose tissue in Holstein-Friesian bulls**. *BMC GENOMICS* 2018, **19**(1):281.

29. Crocker CH, Cammack KM, Hales KE, Freetly HC, Lindholm-Perry AK: **Differential transcript abundance in adipose tissue of mature beef cows during feed restriction and realimentation**. *PLOS ONE* 2018, **13**(3):e194104.

30. Cochran SD, Cole JB, Null DJ, Hansen PJ: **Discovery of single nucleotide polymorphisms in candidate genes associated with fertility and production traits in Holstein cattle**. *BMC GENET* 2013, **14**:49.

31. Poleti MD, DeRijk RH, Rosa AF, Moncau CT, Oliveira PS, Coutinho LL, Eler JP, Balieiro JCC: **Genetic variants in glucocorticoid and mineralocorticoid receptors are associated with concentrations of plasma cortisol, muscle glycogen content, and meat quality traits in male Nellore cattle**. *DOMEST ANIM ENDOCRIN* 2015, **51**:105-113.

32. Majewska M, Lee HY, Tasaki Y, Acosta TJ, Szostek AZ, Siemieniuch M, Okuda K, Skarzynski DJ: **Is cortisol a modulator of interferon tau action in the endometrium during early pregnancy in cattle?** *J REPROD IMMUNOL* 2012, **93**(2):82-93.

33. Fontanesi L, Bertolini F, Scotti E, Trevisi P, Buttazzoni L, Dall'Olio S, Davoli R, Bosi P, Russo V: **Polymorphisms in an obesity-related gene (PCSK1) are associated with fat deposition and production traits in Italian heavy pigs**. *ANIMAL* 2012, **6**(12):1913-1924.

34. Shan L, Sun J, Zhang C, Fang X, Lei C, Lan X, Chen H: **The polymorphisms of bovine PCSK1 gene and their associations with growth traits**. *GENES GENOM* 2011, **33**(1):57-63.

35. Sun J, Shan L, Zhang C, Chen H: **Haplotype combination of the bovine PCSK1 gene sequence variants and association with growth traits in Jiaxian cattle**. *J GENET* 2014, **93**(3):e123-e129.

36. Thatcher WW: **A 100-Year Review: Historical development of female reproductive physiology in dairy cattle1**. *J DAIRY SCI* 2017, **100**(12):10272-10291.

37. Bongiorni S, Gruber CE, Bueno S, Chillemi G, Ferre F, Failla S, Moioli B, Valentini A: **Transcriptomic investigation of meat tenderness in two Italian cattle breeds**. *ANIM GENET* 2016, **47**(3):273-287.

38. C SJ, L RJ, A P: **Mutations in Serac1 or Synj2 cause proximal t haplotype-mediated male mouse sterility but not transmission ratio distortion**. *Proceedings of the National Academy of Sciences* 2005, **102**(9):3342-3347.

39. Tsuda K, Kawahara-Miki R, Sano S, Imai M, Noguchi T, Inayoshi Y, Kono T: **Abundant sequence divergence in the native Japanese cattle Mishima-Ushi (Bos taurus) detected using whole-genome sequencing**. *GENOMICS* 2013, **102**(4):372-378.

40. Peletto S, Strillacci MG, Capucchio MT, Biasibetti E, Modesto P, Acutis PL, Bagnato A: **Genetic basis of Lipomatous Myopathy in Piedmontese beef cattle**. *LIVEST SCI* 2017, **206**:9-16.

41. Chen X, Cheng Z, Zhang S, Werling D, Wathes DC: **Combining Genome Wide Association Studies and Differential Gene Expression Data Analyses Identifies Candidate Genes Affecting Mastitis Caused by Two Different Pathogens in the Dairy Cow**. *Open Journal of Animal Sciences* 2015, **5**(4):358-393.

42. White SN, Mousel MR, Herrmann-Hoesing LM, Reynolds JO, Leymaster KA, Neibergs HL, Lewis GS, Knowles DP: **Genome-wide association identifies multiple genomic regions associated with susceptibility to and control of ovine lentivirus**. *PLOS ONE* 2012, **7**(10):e47829.

43. Mokry FB, Higa RH, de Alvarenga MM, Oliveira DLA, Meirelles SL, Barbosa DSM, Cardoso FF, Morgado DOM, Urbinati I, Meo NS *et al*: **Genome-wide association study for backfat thickness in Canchim beef cattle using Random Forest approach**. *BMC GENET* 2013, **14**:47.

44. Murata T, Yamashiro Y, Kondo T, Nakaichi M, Une S, Taura Y: **Nucleotide sequence of complementary DNA encoding for quaking protein of cow, horse and pig**. *DNA Seq* 2005, **16**(4):300-303.

45. Dos SF, Peixoto MG, Fonseca PA, Pires MF, Ventura RV, Rosse ID, Bruneli FA, Machado MA, Carvalho MR: **Identification of Candidate Genes for Reactivity in Guzerat (Bos indicus) Cattle: A Genome-Wide Association Study**. *PLOS ONE* 2017, **12**(1):e169163.

46. Wang YH, Reverter A, Kemp D, McWilliam SM, Ingham A, Davis CA, Moore RJ, Lehnert SA: **Gene expression profiling of Hereford Shorthorn cattle following challenge with Boophilus microplus tick larvae**. *Australian Journal of Experimental Agriculture* 2007, **47**(12):1397.

47. Ogorevc J, Kunej T, Razpet A, Dovc P: **Database of cattle candidate genes and genetic markers for milk production and mastitis**. *ANIM GENET* 2009, **40**(6):832-851.

48. Speidel SE, Buckley BA, Boldt RJ, Enns RM, Lee J, Spangler ML, Thomas MG: **Genome-wide association study of Stayability and Heifer Pregnancy in Red Angus cattle**. *J ANIM SCI* 2018, **96**(3):846-853.

49. Parker GK, Megonigal JJ, Clay JS, Wolfe CW: **Genome-wide association study for ketosis in US Jerseys using producer-recorded data**. *J DAIRY SCI* 2018, **101**(1):413-424.

50. Qanbari S: **Study of Genomic Structure and Signatures of Recent Positive Selection in Cattle**. *Journal Article; Research Support, Non-U.S. Gov't; Research Support, U.S. Gov't, Non-P.H.S.*: Georg-August University; 2009.

51. Ling YH, Quan Q, Xiang H, Zhu L, Chu MX, Zhang XR, Han CY: **Expression profiles of differentially expressed genes affecting fecundity in goat ovarian tissues**. *Genet Mol Res* 2015, **14**(4):18743-18752.

52. Peters M, Saare M, Kaart T, Haller-Kikkatalo K, Lend AK, Punab M, Metspalu A, Salumets A: **Analysis of polymorphisms in the SRD5A2 gene and semen parameters in Estonian men**. *J Androl* 2010, **31**(4):372-378.

53. Mehla K, Magotra A, Choudhary J, Singh AK, Mohanty AK, Upadhyay RC, Srinivasan S, Gupta P, Choudhary N, Antony B *et al*: **Genome-wide analysis of the heat stress response in Zebu (Sahiwal) cattle**. *GENE* 2014, **533**(2):500-507.

54. Rezende FM, Dietsch GO, Penagaricano F: **Genetic dissection of bull fertility in US Jersey dairy cattle**. *ANIM GENET* 2018, **49**(5):393-402.

55. Kong RS, Liang G, Chen Y, Stothard P, Guan LL: **Transcriptome profiling of the rumen epithelium of beef cattle differing in residual feed intake**. *BMC GENOMICS* 2016, **17**:592.

56. Toro OA, Da SFR, Vercesi FA, Cyrillo J, Zerlotti MM, Curi RA, Ii DVSJ: **Genome-wide identification of runs of homozygosity islands in the Gyr breed (Bos indicus)**. *REPROD DOMEST ANIM* 2020, **55**(3):333-342.

57. Kommadath A, Te PM, Smits MA: **Gene coexpression network analysis identifies genes and biological processes shared among anterior pituitary and brain areas that affect estrous behavior in dairy cows**. *J DAIRY SCI* 2013, **96**(4):2583-2595.

58. Venturini GC, Cardoso DF, Baldi F, Freitas AC, Aspilcueta-Borquis RR, Santos DJA, Camargo GMF, Stafuzza NB, Albuquerque LG, Tonhati H: **Association between single-nucleotide polymorphisms and milk production traits in buffalo**. *Genetics and molecular research : GMR* 2014, **13**(4):10256-10268.

59. Kim SJ, Ka S, Ha JW, Kim J, Yoo D, Kim K, Lee HK, Lim D, Cho S, Hanotte O *et al*: **Cattle genome-wide analysis reveals genetic signatures in trypanotolerant N'Dama**. *BMC GENOMICS* 2017, **18**(1):371.

60. Yi G, Shen M, Yuan J, Sun C, Duan Z, Qu L, Dou T, Ma M, Lu J, Guo J *et al*: **Genome-wide association study dissects genetic architecture underlying longitudinal egg weights in chickens**. *BMC GENOMICS* 2015, **16**:746.

61. Rivera RM: **Consequences of assisted reproductive techniques on the embryonic epigenome in cattle**. *Reproduction, Fertility and Development* 2020, **32**(2):65.

62. Lai FN, Zhai HL, Cheng M, Ma JY, Cheng SF, Ge W, Zhang GL, Wang JJ, Zhang RQ, Wang X *et al*: **Whole-genome scanning for the litter size trait associated genes and SNPs under selection in dairy goat (Capra hircus)**. *Sci Rep* 2016, **6**:38096.

63. Zhang Y, Dean C, Chessum L, Nguyen D, Stewart M, Taylor M, Cookson WO, Moffatt MF: **Functional analysis of a novel ENU-induced PHD finger 11 (Phf11) mouse mutant**. *MAMM GENOME* 2014, **25**(11-12):573-582.

64. Larska M, Polak MP, Zmudzinski JF, Torres JM: **Comparison of mRNA expression levels of selected genes in the brain stem of cattle naturally infected with classical and atypical BSE**. *BRAIN RES* 2010, **1351**:13-22.

65. Delesque-Touchard N, Pendaries C, Volle-Challier C, Millet L, Salel V, Herve C, Pflieger AM, Berthou-Soulie L, Prades C, Sorg T *et al*: **Regulator of G-protein signaling 18 controls both platelet generation and function**. *PLOS ONE* 2014, **9**(11):e113215.

66. Lee H, Jaffe AE, Feinberg JI, Tryggvadottir R, Brown S, Montano C, Aryee MJ, Irizarry RA, Herbstman J, Witter FR *et al*: **DNA methylation shows genome-wide association of NFIX, RAPGEF2 and MSRB3 with gestational age at birth**. *INT J EPIDEMIOL* 2012, **41**(1):188-199.

67. Tang F, Wang B, Li N, Wu Y, Jia J, Suo T, Chen Q, Liu YJ, Tang J: **RNF185, a novel mitochondrial ubiquitin E3 ligase, regulates autophagy through interaction with BNIP1**. *PLOS ONE* 2011, **6**(9):e24367.

68. Richardson IW, Berry DP, Wiencko HL, Higgins IM, More SJ, McClure J, Lynn DJ, Bradley DG: **A genome-wide association study for genetic susceptibility to Mycobacterium bovis infection in dairy cattle identifies a susceptibility QTL on chromosome 23**. *GENET SEL EVOL* 2016, **48**:19.

69. Kramer LM, Ghaffar MA, Koltes JE, Fritz-Waters ER, Mayes MS, Sewell AD, Weeks NT, Garrick DJ, Fernando RL, Ma L *et al*: **Epistatic interactions associated with fatty acid concentrations of beef from angus sired beef cattle**. *BMC GENOMICS* 2016, **17**(1):891.

70. Alluwaimi AM, Badi FA: **Mycobacterium avium subspecies paratuberculosis infection in naturally infected cattle is associated with an upregulation of lipid metabolism gene expression**. *International Journal of Advanced Life Sciences* 2015, **8**(4).

71. Wu X, Fang M, Liu L, Wang S, Liu J, Ding X, Zhang S, Zhang Q, Zhang Y, Qiao L *et al*: **Genome wide association studies for body conformation traits in the Chinese Holstein cattle population**. *BMC GENOMICS* 2013, **14**:897.

72. Mei C, Wang H, Liao Q, Khan R, Raza S, Zhao C, Wang H, Cheng G, Tian W, Li Y *et al*: **Genome-wide analysis reveals the effects of artificial selection on production and meat quality traits in Qinchuan cattle**. *GENOMICS* 2019, **111**(6):1201-1208.

73. Taye M, Lee W, Caetano-Anolles K, Dessie T, Hanotte O, Mwai OA, Kemp S, Cho S, Oh SJ, Lee HK *et al*: **Whole genome detection of signature of positive selection in African cattle reveals selection for thermotolerance**. *ANIM SCI J* 2017, **88**(12):1889-1901.

74. Wijga S, Bastiaansen JW, Wall E, Strandberg E, de Haas Y, Giblin L, Bovenhuis H: **Genomic associations with somatic cell score in first-lactation Holstein cows**. *J DAIRY SCI* 2012, **95**(2):899-908.

75. Alshawi A, Essa A, Al-Bayatti S, Hanotte O: **Genome Analysis Reveals Genetic Admixture and Signature of Selection for Productivity and Environmental Traits in Iraqi Cattle**. *FRONT GENET* 2019, **10**:609.

76. Lindholm Perry AK, Cunningham HC, Kuehn LA, Vallet JL, Keele JW, Foote AP, Cammack KM, Freetly HC: **Relationships between the genes expressed in the mesenteric adipose tissue of beef cattle and feed intake and gain1**. *ANIM GENET* 2017, **48**(4):386-394.

77. Fink S, Momke S, Wohlke A, Distl O: **Genes on bovine chromosome 18 associated with bilateral convergent strabismus with exophthalmos in German Brown cattle**. *MOL VIS* 2008, **14**:1737-1751.

78. Fink S, Momke S, Distl O: **PLXNC1 and RDH13 associated with bilateral convergent strabismus with exophthalmus in German Brown cattle**. *MOL VIS* 2012, **18**:2229-2240.

79. Zhou C, Shen D, Li C, Cai W, Liu S, Yin H, Shi S, Cao M, Zhang S: **Comparative Transcriptomic and Proteomic Analyses Identify Key Genes Associated With Milk Fat Traits in Chinese Holstein Cows**. *FRONT GENET* 2019, **10**:672.

80. Abo-Ismail MK, Brito LF, Miller SP, Sargolzaei M, Grossi DA, Moore SS, G P, P S, S N, Schenkel FS: **Genome wide association studies and genomic prediction of breeding values for calving performance and body conformation traits in Holstein cattle**. *GENET SEL EVOL* 2017, **49**(1):82.

81. Anastasaki C, Dahiya S, Gutmann DH: **KIR2DL5 mutation and loss underlies sporadic dermal neurofibroma pathogenesis and growth**. *Oncotarget* 2017, **8**(29):47574.

82. Guethlein LA, Abi-Rached L, Hammond JA, Parham P: **The expanded cattle KIR genes are orthologous to the conserved single-copy KIR3DX1 gene of primates**. *IMMUNOGENETICS* 2007, **59**(6):517-522.

83. Halfteck GG, Elboim M, Gur C, Achdout H, Ghadially H, Mandelboim O: **Enhanced in vivo growth of lymphoma tumors in the absence of the NK-activating receptor NKp46/NCR1**. *J IMMUNOL* 2009, **182**(4):2221-2230.

84. Diogenes MN, Guimaraes AL, Leme LO, Dode MA: **Bovine in vitro embryo production: the effects of fibroblast growth factor 10 (FGF10)**. *J Assist Reprod Genet* 2017, **34**(3):383-390.

85. Gasperin BG, Ferreira R, Rovani MT, Santos JT, Buratini J, Price CA, Gonçalves PBD: **FGF10 inhibits dominant follicle growth and estradiol secretion in vivo in cattle**. *REPRODUCTION* 2012, **143**(6):815-823.

86. Castilho ACS, Price CA, Dalanezi F, Ereno RL, Machado MF, Barros CM, Gasperin BG, Gonçalves PBD, Buratini J: **Evidence that fibroblast growth factor 10 plays a role in follicle selection in cattle**. *Reproduction, Fertility and Development* 2017, **29**(2):234.

87. Igoshin AV, Yurchenko AA, Belonogova NM, Petrovsky DV, Aitnazarov RB, Soloshenko VA, Yudin NS, Larkin DM: **Genome-wide association study and scan for signatures of selection point to candidate genes for body temperature maintenance under the cold stress in Siberian cattle populations**. *BMC GENET* 2019, **20**(Suppl 1):26.

88. Yodklaew P, Koonawootrittriron S, Elzo MA, Suwanasopee T, Laodim T: **Genome-wide association study for lactation characteristics, milk yield and age at first calving in a Thai multibreed dairy cattle population**. *Agriculture and Natural Resources* 2017, **51**(3):223-230.

89. Kandaswamy R, McQuillin A, Curtis D, Gurling H: **Allelic association, DNA resequencing and copy number variation at the metabotropic glutamate receptor GRM7 gene locus in bipolar disorder**. *Am J Med Genet B Neuropsychiatr Genet* 2014, **165B**(4):365-372.

90. Koh YQ, Peiris HN, Vaswani K, Almughlliq FB, Meier S, Burke CR, Roche JR, Reed CB, Arachchige BJ, Reed S *et al*: **Proteome profiling of exosomes derived from plasma of heifers with divergent genetic merit for fertility**. *J DAIRY SCI* 2018, **101**(7):6462-6473.

91. Bessho T, Okada T, Kimura C, Shinohara T, Tomiyama A, Imamura A, Kuwamura M, Nishimura K, Fujimori K, Shuto S *et al*: **Novel Characteristics of Trypanosoma brucei Guanosine 5'-monophosphate Reductase Distinct from Host Animals**. *PLoS Negl Trop Dis* 2016, **10**(1):e4339.

92. Moreno-Sánchez N, Rueda J, Carabaño MJ, Reverter A, McWilliam S, González C, Díaz C: **Skeletal muscle specific genes networks in cattle**. *FUNCT INTEGR GENOMIC* 2010, **10**(4):609-618.

93. Verma P, Sharma A, Sodhi M, Thakur K, Kataria RS, Niranjan SK, Bharti VK, Kumar P, Giri A, Kalia S *et al*: **Transcriptome Analysis of Circulating PBMCs to Understand Mechanism of High Altitude Adaptation in Native Cattle of Ladakh Region**. *Sci Rep* 2018, **8**(1):7681.

94. Zhu X, Topouzis S, Liang LF, Stotish RL: **Myostatin signaling through Smad2, Smad3 and Smad4 is regulated by the inhibitory Smad7 by a negative feedback mechanism**. *CYTOKINE* 2004, **26**(6):262-272.

95. Raychaudhuri S: **MicroRNAs overexpressed in growth-restricted rat skeletal muscles regulate the glucose transport in cell culture targeting central TGF-beta factor SMAD4**. *PLOS ONE* 2012, **7**(4):e34596.

96. Liao X, Lan C, Liao D, Tian J, Huang X: **Exploration and detection of potential regulatory variants in refractive error GWAS**. *Sci Rep* 2016, **6**:33090.

97. Iso-Touru T, Sahana G, Guldbrandtsen B, Lund MS, Vilkki J: **Genome-wide association analysis of milk yield traits in Nordic Red Cattle using imputed whole genome sequence variants**. *BMC GENET* 2016, **17**:55.

98. Cai Z, Guldbrandtsen B, Lund MS, Sahana G: **Prioritizing candidate genes post-GWAS using multiple sources of data for mastitis resistance in dairy cattle**. *BMC GENOMICS* 2018, **19**(1):656.

99. Zhang W, Yang F, Zhu Z, Yang Y, Wang Z, Cao W, Dang W, Li L, Mao R, Liu Y *et al*: **Cellular DNAJA3, a Novel VP1-Interacting Protein, Inhibits Foot-and-Mouth Disease Virus Replication by Inducing Lysosomal Degradation of VP1 and Attenuating Its Antagonistic Role in the Beta Interferon Signaling Pathway**. *J VIROL* 2019, **93**(13).

100. Rossetti CA, Galindo CL, Everts RE, Lewin HA, Garner HR, Adams LG: **Comparative analysis of the early transcriptome of Brucella abortus – Infected monocyte-derived macrophages from cattle naturally resistant or susceptible to brucellosis**. *RES VET SCI* 2011, **91**(1):40-51.

101. Abdollahi-Arpanahi R, Carvalho MR, Ribeiro ES, Peñagaricano F: **Association of lipid-related genes implicated in conceptus elongation with female fertility traits in dairy cattle**. *J DAIRY SCI* 2019, **102**(11):10020-10029.

102. Carvalho ME, Baldi FS, Santana MHA, Ventura RV, Oliveira GA, Bueno RS, Bonin MN, Rezende FM, Coutinho LL, Eler JP *et al*: **Identification of genomic regions related to tenderness in Nellore beef cattle**. *Advances in Animal Biosciences* 2017, **8**(s1):s42-s44.

103. Han Y, Peñagaricano F: **Unravelling the genomic architecture of bull fertility in Holstein cattle**. *BMC GENET* 2016, **17**(1):143.

104. E GX, Duan XH, Zhang JH, Huang YF, Zhao YJ, Na RS, Zhao ZQ, Ma YH, Chu MX, Basang WD *et al*: **Genome-wide selection signatures analysis of litter size in Dazu black goats using single-nucleotide polymorphism**. *3 BIOTECH* 2019, **9**(9):336.

105. Mastranestasis I, Kominakis A, Hager-Theodorides AL, Ekateriniadou LV, Ligda C, Theodorou K: **Associations between genetic polymorphisms and phenotypic traits in the Lesvos dairy sheep**. *SMALL RUMINANT RES* 2016, **144**:205-210.

106. Freidin MB, Bragina E, Fedorova OS, Deev IA, Kulikov ES, Ogorodova LM, Puzyrev VP: **Genome-wide association study of allergic diseases in Russians of Western Siberia**. *Mol Biol (Mosk)* 2011, **45**(3):464-472.

107. Ryu J, Lee C: **Genetic association of marbling score with intragenic nucleotide variants at selection signals of the bovine genome**. *ANIMAL* 2016, **10**(4):566-570.

108. Ryu J, Lee C: **Identification of contemporary selection signatures using composite log likelihood and their associations with marbling score in Korean cattle**. *ANIM GENET* 2014, **45**(6):765-770.

109. Sorbolini S, Marras G, Gaspa G, Dimauro C, Cellesi M, Valentini A, Macciotta NP: **Detection of selection signatures in Piemontese and Marchigiana cattle, two breeds with similar production aptitudes but different selection histories**. *GENET SEL EVOL* 2015, **47**:52.

110. Howard JT, Pryce JE, Haile-Mariam M, Maltecca C: **REGIONS IMPACTING INBREEDING DEPRESSION AND THEIR ASSOCIATION WITH ADDITIVE GENETIC EFFECTS FOR JERSEY CATTLE FROM THE UNITED STATES OF AMERICA AND AUSTRALIA**. In*.*; 2015.

111. Hardie LC, VandeHaar MJ, Tempelman RJ, Weigel KA, Armentano LE, Wiggans GR, Veerkamp RF, de Haas Y, Coffey MP, Connor EE *et al*: **The genetic and biological basis of feed efficiency in mid-lactation Holstein dairy cows**. *J DAIRY SCI* 2017, **100**(11):9061-9075.

112. Strillacci MG, Frigo E, Canavesi F, Ungar Y, Schiavini F, Zaniboni L, Reghenzani L, Cozzi MC, Samor AB, Kashi Y *et al*: **Quantitative trait loci mapping for conjugated linoleic acid, vaccenic acid and Δ 9-desaturase in Italian Brown Swiss dairy cattle using selective DNA pooling**. *ANIM GENET* 2014, **45**(4):485-499.

113. Dadousis C, Pegolo S, Rosa G, Gianola D, Bittante G, Cecchinato A: **Pathway-based genome-wide association analysis of milk coagulation properties, curd firmness, cheese yield, and curd nutrient recovery in dairy cattle**. *J DAIRY SCI* 2017, **100**(2):1223-1231.

114. Chen Q, Zhan J, Wang J, Qu K, Zhang F, Shen J, Jia P, Ning Q, Zhang J, Chen N *et al*: **Whole-genome analyses identify loci and selective signals associated with body size in cattle**. *J ANIM SCI* 2020.

115. Cole JB, Wiggans GR, Ma L, Sonstegard TS, Lawlor TJ, Crooker BA, Van Tassell CP, Yang J, Wang S, Matukumalli LK *et al*: **Genome-wide association analysis of thirty one production, health, reproduction and body conformation traits in contemporary U.S. Holstein cows**. *BMC GENOMICS* 2011, **12**(1):408.

116. Shimizu T, Krebs S, Bauersachs S, Blum H, Wolf E, Miyamoto A: **Actions and interactions of progesterone and estrogen on transcriptome profiles of the bovine endometrium**. *PHYSIOL GENOMICS* 2010, **42A**(4):290-300.

117. Silva T, Santos EC, Annes K, Soares CA, Leite RF, Lima CB, Milazzotto MP: **Morphokinetic-related response to stress in individually cultured bovine embryos**. *THERIOGENOLOGY* 2016, **86**(5):1308-1317.

118. Rola-Luszczak M, Materniak M, Pluta A, Hulst M, Kuzmak J: **Transcriptomic microarray analysis of BoMac cells after infection with bovine foamy virus**. *ARCH VIROL* 2014, **159**(6):1515-1519.

119. Srikanth K, Kwon A, Lee E, Chung H: **Characterization of genes and pathways that respond to heat stress in Holstein calves through transcriptome analysis**. *Cell Stress Chaperones* 2017, **22**(1):29-42.

120. Han HI, Lee SH, Song EJ, Lee S, Cheong HT, Yang BK, Park CK: **Effect of uterine histotroph on embryo development in pigs**. *한국동물생명공학회지 (구 한국수정란이식학회지)* 2016, **31**(3):199-205.

121. Benoit G, Warma A, Lussier JG, Ndiaye K: **Gonadotropin regulation of ankyrin-repeat and SOCS-box protein 9 (ASB9) in ovarian follicles and identification of binding partners**. *PLOS ONE* 2019, **14**(2):e212571.

122. Ovilo C, Benitez R, Fernandez A, Nunez Y, Ayuso M, Fernandez AI, Rodriguez C, Isabel B, Rey AI, Lopez-Bote C *et al*: **Longissimus dorsi transcriptome analysis of purebred and crossbred Iberian pigs differing in muscle characteristics**. *BMC GENOMICS* 2014, **15**:413.
