# Supplementary material for: Genetic Parameters and Genome-Wide Association Studies of Eight Longevity Traits Representing Either Full or Partial Lifespan in Chinese Holsteins
Source: Front Genet. 2021 Feb 25;12:634986. doi: 10.3389/fgene.2021.634986 (PMC7947242; doi:10.3389/fgene.2021.634986)
Supplement: Supplementary Table 3 — Number of SNPs and genotyped animals kept for association analysis on each longevity trait in Chinese Holstein cattle. [file Data_Sheet_3.docx]

**SUPPLEMENTARY MATERIAL 3**

Supplementary Table 3. Number of SNPs and genotyped animals kept for association analysis on each longevity trait in Chinese Holstein cattle

| Traits^1^ | N. of animals | N. of SNPs |
| --- | --- | --- |
| HL | 2,592 | 116,547 |
| PL | 2,466 | 116,563 |
| ML | 2,584 | 116,557 |
| Lon11 | 2,474 | 116,559 |
| Lon12 | 2,476 | 116,570 |
| Lon13 | 2,465 | 116,563 |
| Lon14 | 2,461 | 116,550 |
| Lon15 | 2,463 | 116,557 |

^1^Lon11, the days from the first calving to the end of the first lactation or culling; Lon12, the days from the first calving to the end of the second lactation or culling; Lon13, the days from the first calving to the end of the third lactation or culling; Lon14, the days from the first calving to the end of the fourth lactation or culling; Lon15, the days from the first calving to the end of the fifth lactation or culling; PL, productive life referring the days from the first calving to culling or dead; ML, milking life referring the days from the first calving to culling or death but excludes all dry periods; HL, herd life referring the days from birth to culling or death.

Supplementary Table 4. Descriptive statistics of de-regressed estimate breeding values for longevity traits in Chinese Holsteins

| Trait^1^ | N | Mean | Min | Max |
| --- | --- | --- | --- | --- |
| HL | 2,592 | 7.96 | -99.57 | 104.11 |
| PL | 2,466 | 3.17 | -159.13 | 154.81 |
| ML | 2,584 | 10.38 | -93.02 | 98.62 |
| Lon11 | 2,474 | 5.51 | -25.88 | 29.73 |
| Lon12 | 2,476 | 1.85 | -69.52 | 78.94 |
| Lon13 | 2,465 | -1.83 | -88.76 | 87.58 |
| Lon14 | 2,461 | -2.99 | -109.79 | 108.34 |
| Lon15 | 2,463 | -10.84 | -117.87 | 113.61 |

^1^Lon11, the days from the first calving to the end of the first lactation or culling; Lon12, the days from the first calving to the end of the second lactation or culling; Lon13, the days from the first calving to the end of the third lactation or culling; Lon14, the days from the first calving to the end of the fourth lactation or culling; Lon15, the days from the first calving to the end of the fifth lactation or culling; PL, productive life referring the days from the first calving to culling or dead; ML, milking life referring the days from the first calving to culling or death but excludes all dry periods; HL, herd life referring the days from birth to culling or death.
